# Cisplatin prevents breast cancer metastasis through blocking early EMT and retards cancer growth together with paclitaxel

**DOI:** 10.7150/thno.46460

**Published:** 2021-01-01

**Authors:** Haitao Wang, Sen Guo, Seung-Jin Kim, Fangyuan Shao, Joshua Wing Kei Ho, Kuan Un Wong, Zhengqiang Miao, Dapeng Hao, Ming Zhao, Jun Xu, Jianming Zeng, Koon Ho Wong, Lijun Di, Ada Hang-Heng Wong, Xiaoling Xu, Chu-Xia Deng

**Affiliations:** 1 Cancer Center, Faculty of Health Sciences, University of Macau, Macau SAR, China.; 2 Center for Precision Medicine Research and Training, University of Macau, Macau SAR, China.; 3 Genetics of Development and Disease Branch, 10/9N105, National Institute of Diabetes, Digestive and Kidney Diseases, National Institutes of Health, Bethesda, MD, 20892, USA.; 4 School of Biomedical Sciences, Li Ka Shing Faculty of Medicine, The University of Hong Kong, Hong Kong SAR, China.; 5 Institute of Translational Medicine, Faculty of Health Sciences, University of Macau, Macau SAR, China.

**Keywords:** cisplatin, paclitaxel, neoadjuvant therapy, TGFβ, metastasis

## Abstract

Cancer growth is usually accompanied by metastasis which kills most cancer patients. Here we aim to study the effect of cisplatin at different doses on breast cancer growth and metastasis.

**Methods:** We used cisplatin to treat breast cancer cells, then detected the migration of cells and the changes of epithelial-mesenchymal transition (EMT) markers by migration assay, Western blot, and immunofluorescent staining. Next, we analyzed the changes of RNA expression of genes by RNA-seq and confirmed the binding of activating transcription factor 3 (ATF3) to cytoskeleton related genes by ChIP-seq. Thereafter, we combined cisplatin and paclitaxel in a neoadjuvant setting to treat xenograft mouse models. Furthermore, we analyzed the association of disease prognosis with cytoskeletal genes and ATF3 by clinical data analysis.

**Results:** When administered at a higher dose (6 mg/kg), cisplatin inhibits both cancer growth and metastasis, yet with strong side effects, whereas a lower dose (2 mg/kg) cisplatin blocks cancer metastasis without obvious killing effects. Cisplatin inhibits cancer metastasis through blocking early steps of EMT. It antagonizes transforming growth factor beta (TGFβ) signaling through suppressing transcription of many genes involved in cytoskeleton reorganization and filopodia formation which occur early in EMT and are responsible for cancer metastasis. Mechanistically, TGFβ and fibronectin-1 (FN1) constitute a positive reciprocal regulation loop that is critical for activating TGFβ/SMAD3 signaling, which is repressed by cisplatin induced expression of ATF3. Furthermore, neoadjuvant administration of cisplatin at 2 mg/kg in conjunction with paclitaxel inhibits cancer growth and blocks metastasis without causing obvious side effects by inhibiting colonization of cancer cells in the target organs.

**Conclusion:** Thus, cisplatin prevents breast cancer metastasis through blocking early EMT, and the combination of cisplatin and paclitaxel represents a promising therapy for killing breast cancer and blocking tumor metastasis.

## Introduction

Cancer metastasis is the spread of primary tumor cells to distant sites of the body 1. Deaths caused by cancer metastasis accounts for the majority of all cancer deaths although with wide variations in different countries 2, 3. Cancer metastasis involves a consecutive physiological process including cancer cells invade through extracellular matrix (ECM), enter into the circulation (intravasation), survive within the blood circulation or lymph system, disseminate into distant tissues (extravasation), and finally colonize and grow as secondary tumors 4-6. Numerous experimental data indicated that the epithelial-to-mesenchymal transition (EMT) promotes tumor metastasis, yet this notion was challenged by several recent studies 7-11. EMT is a multi-step process, in which epithelial cells gradually lose cell-cell adhesion, undergo extensive cytoskeleton reorganization, change their cellular morphology and become migratory and invasive mesenchymal cells 2, 5, 12-14. Many factors are involved in triggering EMT, including hypoxia 15, extracellular acidosis 16, succinate dehydrogenase (SDH) inhibition 17, and signaling cascade mediated by transforming growth factor beta (TGFβ) 5, 18.

TGFβ is a multi-functional cytokine in mammals, consisting of three isoforms, i.e. TGFβ1, TGFβ2 and TGFβ3 5, 19. The activation of TGFβ is initiated by its interaction with latent TGFβ binding proteins (LTBPs) in the extracellular matrix 20. The activated TGFβ then binds to its membrane receptor II (TGFβ-RII), which, in turn, forms hetero-complex with TGFβ-RI, leading to autophosphorylation of TGFβ-RI. This complex then phosphorylates the SMAD2/3 proteins in the cytoplasm, followed by recruitment of SMAD4 to form SMAD2/3/4 complex, which is transferred into the nucleus where it activates transcription of TGFβ-downstream genes 21, 22. This action results in activation of signaling cascades that trigger multiple cellular processes, including ECM protein production, cytoskeleton reorganization leading to cell adherence changes, cell movement, cancer metastasis, *etc* 23-25.

Cisplatin belongs to a class of platinum drugs that alkylates DNA through formation of platinum-DNA adducts, leading to DNA damage, G1/S arrest and apoptosis 26, 27. Like all other platinum drugs, cisplatin is initially effective in killing cancer cells, but resistance eventually develops 26-30. Even with side effects, cisplatin is still used in initial clinical therapies and administered to recurrent and/or metastatic cancer patients 31-33. We have previously observed that treatment of murine mammary tumors with cisplatin initially suppressed tumor growth, but drug resistance occurred after prolonged drug exposure, leading to uncontrolled tumor recurrence and growth 34. However, the resistant tumors seemed to proliferate but failed to metastasize in the presence of cisplatin. In addition, cisplatin at a low dose range, 2.5-5 μg/mL, also blocked cell migration that was accompanied by impaired cytoskeleton remodeling, yet the underlying mechanism is not clear 34.

Because TGFβ plays essential roles in inducing cytoskeleton remodeling, and cell movement *in vitro* and cancer metastasis *in vivo* 23, 24, we hypothesized that the inhibition of cisplatin on cytoskeleton remodeling and cancer metastasis might be mediated through antagonizing TGFβ signaling. To investigate this, we focused on the functional interplay between cisplatin and TGFβ during the earliest stages of EMT. Our data uncovers a positive regulation loop among TGFβ and FN1 in maintaining TGFβ activity, which is interrupted by overexpression of ATF3 induced by cisplatin. We also show that in the neoadjuvant treatment setting by which the combined therapy of cisplatin and paclitaxel together inhibits breast cancer growth and metastasis, hence providing a new therapeutic route to treating breast cancer.

## Results

### Cisplatin inhibits the early changes of EMT and blocks cell migration induced by TGFβ

**Figure 1:**
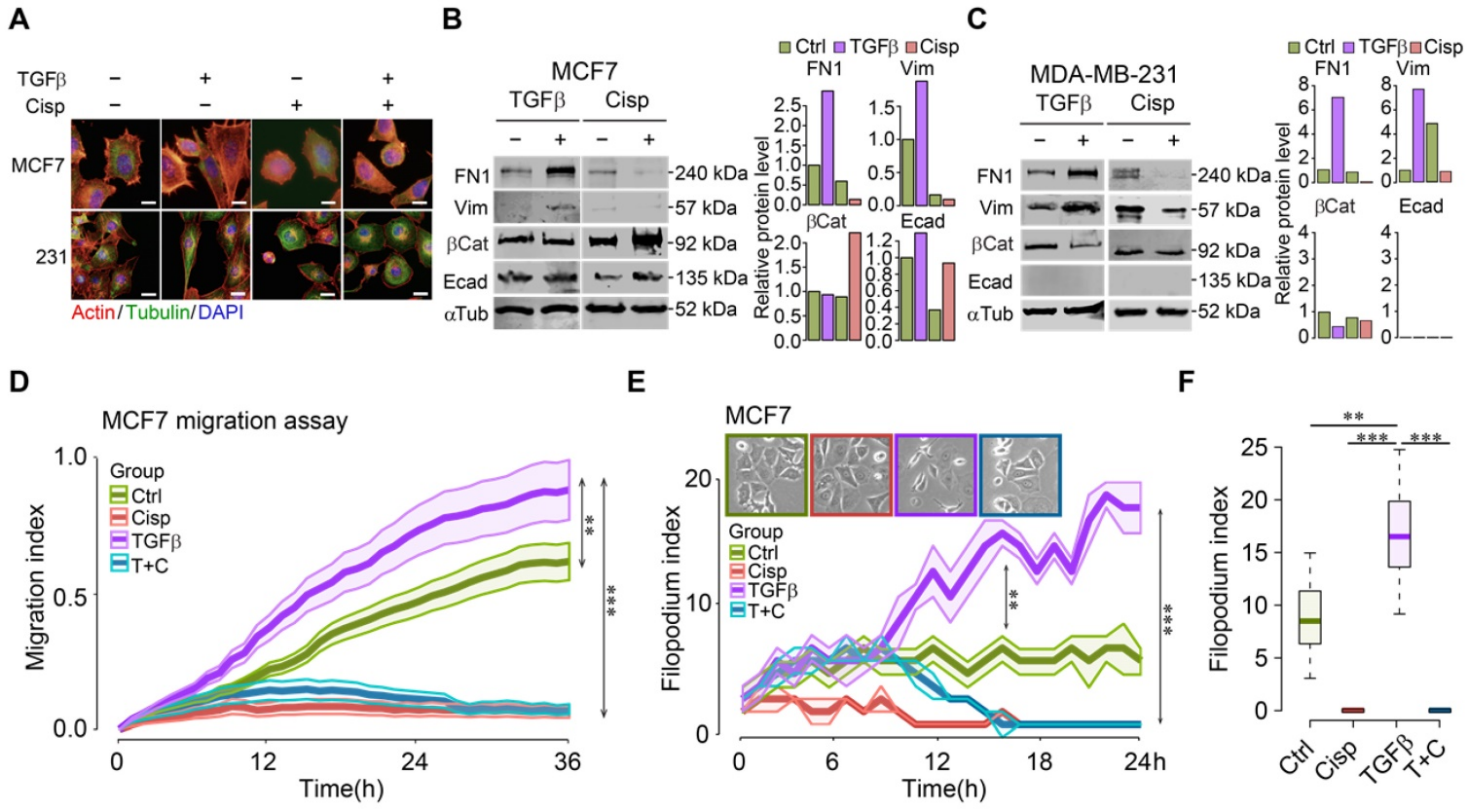
** Cisplatin antagonizes TGFβ-induced EMT and cell movement of breast cancer cells. A** TGFβ induces EMT in MCF7 and MDA-MB-231 cells, which is counteracted by cisplatin. Filamentous actin was stained by 1:1000 phalloidin. Bar = 50 μm. **B**,** C** TGFβ and cisplatin induce expression changes of mesenchymal and epithelial markers triggered in opposite directions revealed by Western blot. Quantification of FN1, Vimentin, β-catenin and E-cadherin levels were shown on the right panels. **D** TGFβ enhances cell migration as compared to control cells whereas cisplatin not only inhibits cell migration, but also overrides the stimulatory effect of TGFβ in MCF7 cells. **E**,** F** Calculation of filopodium index during a 24-h time lapse (**E**) and at 24h point (**F**) reveals that TGFβ significantly increases filopodium formation as compared to control, whereas cisplatin completely blocks filopodium formation in either cisplatin or cisplatin/TGFβ treatment conditions in MCF7 cells. Insert in E shows MCF7 cell morphology at 24h point. In (**D**-**F**), data represent means ± standard deviations (SDs). See also Figure S1, Figure S2 and Figure S3. Concentration of drugs in this figure: TGFβ [5 ng/mL] cisplatin [10 μM]. Immunoblots shown in this figure have 3 replicates. ns, *, ** and *** means not significant, p < 0.05, p < 0.005, and p < 0.0005, respectively.

To understand cell morphological changes during the process of EMT upon drug treatment in cancer cells, we conducted immunofluorescent staining of cells with phalloidin and antibodies against cytoskeletal proteins of tubulin and actin with/without TGFβ. The data showed that TGFβ stimulated stress fibers formation, whereas cisplatin counteracted this phenotype in the absence or presence of TGFβ (Figure 1A). It is noteworthy that cell transformation of the epithelial MCF7 cells under TGFβ treatment was more prominent than the basal-type MDA-MB-231 cells, which inherently display some mesenchymal features. On the other hand, most MDA-MB-231 cells rounded up after cisplatin mono-treatment or TGFβ/cisplatin combined treatment (Figure 1A), reminiscent of the reversal of EMT, which is known as mesenchymal-to-epithelial transition (MET) 14, 35. Molecular analysis revealed that TGFβ treatment upregulated mesenchymal markers such as fibronectin 1 (FN1) and vimentin, while cisplatin treatment, not only inhibited them but also triggered higher levels of epithelial markers such as β-catenin and E-cadherin in MCF7 cells (Figure 1B). In MDA-MB-231 cells, TGFβ upregulated fibronectin 1 (FN1) and vimentin, and inhibited β-catenin, but had no effect on E-cadherin that was not detected (Figure 1C) due to low expression 36. Of note, cisplatin treatment repressed expression of mesenchymal genes induced by TGFβ with no obvious effect on the two epithelial markers studied (Figure 1C).

Next, we monitored real-time cell migration by real-time impedance assay 37. While cell migration was gradually stimulated by TGFβ, decreased cell migration was observed 3 h after cisplatin addition, regardless of the presence or absence of TGFβ in both MCF7 (Figure 1D) and MDA-MB-231 (Figure S1A) cells. This observation suggests that cisplatin blocks cell migration induced by TGFβ at the earliest time point, preceding EMT induced by TGFβ.

In order to confirm that cell migration indeed resulted from increased cell motility, we counted the number of filopodia in these cells after treatment with TGFβ and/or cisplatin as compared to control during a 24-h time course. The analysis indicated that TGFβ significantly increased the number of filopodia 3 h post-treatment and the induction sustained until the end of experiment. Cisplatin treatment effectively blocked filopodia formation in MCF7 cells (Figure 1E-F), and in MDA-MB-231 cells (Figure S1B-C). Because filopodia formation during cell movement is associated with dynamic cytoskeleton rearrangement, we hypothesize that the consequence of cisplatin inhibiting cell migration is associated with its abolishment of cytoskeleton rearrangement induced by TGFβ. Similar effects of TGFβ and cisplatin on morphological changes, gene expression, and migration of a murine cell line, 69 that was derived from a mouse *Brca1* deficient mammary tumor 38, was observed (Figure S2). During time-lapse imaging of 4T1 cell line that was derived from a mammary cancer of BALB/c mouse, we found that most cells underwent EMT induced by TGFβ within 24-36 h, varying on the different density of cells (Figure S3A-B). During an observation period of 6-36 h after TGFβ treatment, we found that although cells gained higher motility 6 h post TGFβ treatment, prolonged treatment enhanced faster migration (Figure S3C-D). The EMT process was completely blocked by cisplatin treatment from the start of the treatment (Figure S3E).

### Cisplatin affects expression of genes that are involved in extracellular matrix and cytoskeleton rearrangement mediated by TGFβ

To decipher the antagonistic effect of cisplatin on TGFβ induced EMT, we performed whole transcriptome RNA sequencing (RNA-seq) under the treatment of cisplatin and/or TGFβ at 4 time points of 0, 6, 12 and 24 h in MCF7 cells.

**Figure 2:**
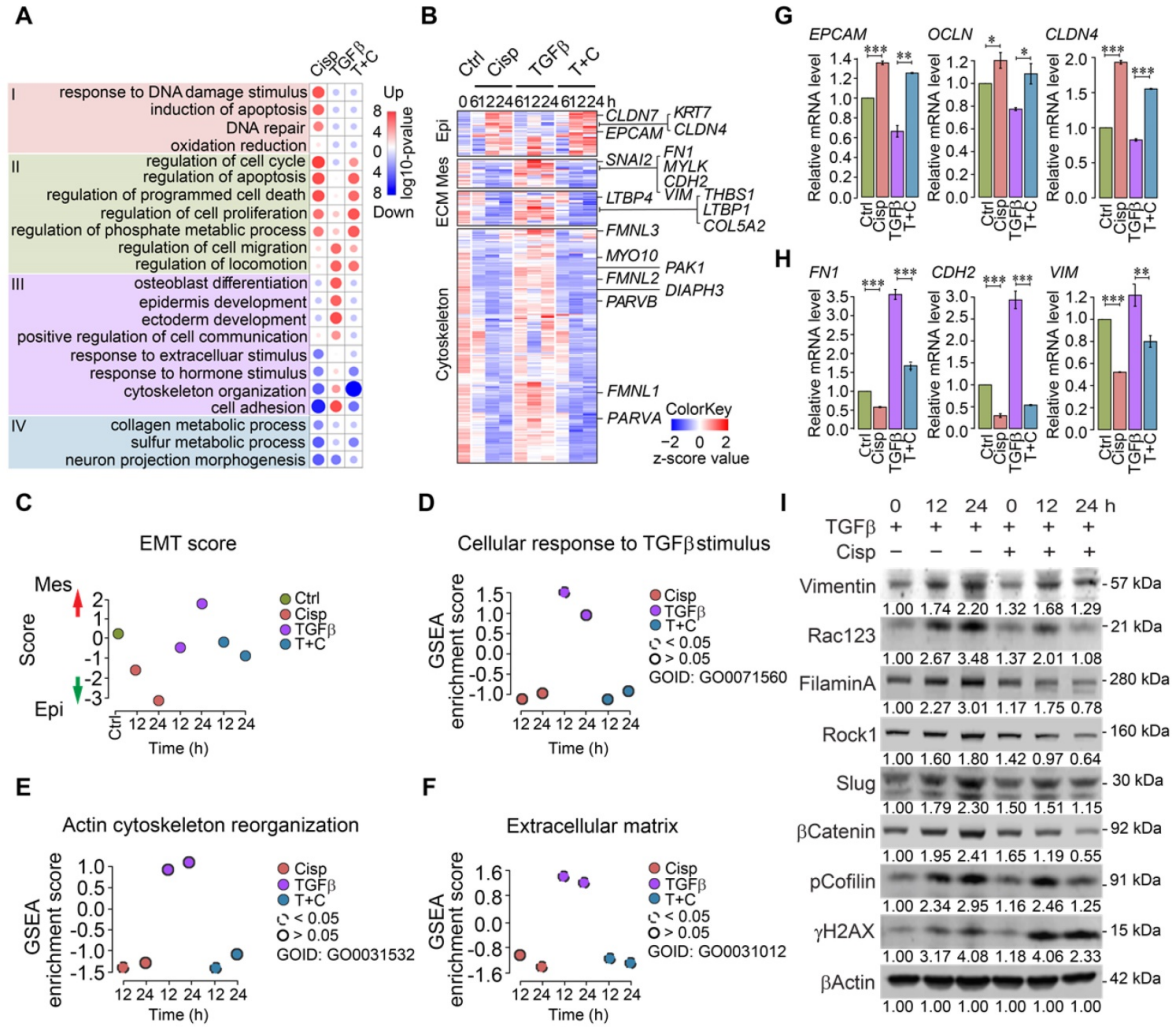
** Analysis of gene expression of MCF7 and MDA-MB-231 cells at different time points after treatment with TGFβ and/or cisplatin. A** Heatmap of overall gene expression in cisplatin and/or TGFβ treated MCF7 cells. **B** Illustration of expression changes of genes involved in epithelial and mesenchymal signatures, ECM function as well as cytoskeleton rearrangement induced by cisplatin and/or TGFβ treatment in MCF7 cells. **C-F** Calculation of EMT score (**C**), GSEA gene enrichment score of TGFβ responding genes (**D**), actin cytoskeleton reorganization (**E**), and extracellular matrix (**F**) in MCF7 cells after single or combined treatment with TGFβ and cisplatin. **G**, **H** RT-qPCR validation of selected epithelial (**G**), and mesenchymal (**H**) marker genes after cisplatin and TGFβ treatment in MCF7 cells. **I** Western blot analysis of mesenchymal marker genes that are induced by TGFβ and inhibited by cisplatin in MDA-MB-231 cells. Quantifications of each proteins are shown under each band. Replicate = 3. In (**G**, **H**), data represent means ± standard deviations (SDs). See also Figure S4. Concentration of drugs in this figure: TGFβ [5 ng/mL] cisplatin [10 μM]. ns, *, ** and *** means not significant, p < 0.05, p < 0.005, and p < 0.0005, respectively.

Gene ontology and pathway analysis of RNA expression profiles of MCF7 cells revealed distinct patterns in different treatment groups at 12 h post-treatment (Figure 2A). Cisplatin treatment induced expression of genes involved in cell cycle, apoptosis, DNA damage response and repair, *etc* (Figure 2A, Group I). While TGFβ treatment exhibited mild effect on the expression of genes in Group I, it elicited stronger effect on expression of genes involved in cell migration, locomotion, and communication, as well as epidermis development, *etc* (Figure 2A, Group II). Meanwhile, cisplatin repressed expression of some genes that could be further divided into 2 groups, i.e. genes whose expression is stimulated by TGFβ treatment (cytoskeleton organization and cell adhesion, Figure 2A, Group III), and genes whose expression is not significantly stimulated by TGFβ (Figure 2A, Group IV). Specifically, our data revealed that cisplatin upregulated a group of marker genes for epithelial cells, and at the same time, it also inhibited expression of genes that are involved in mesenchymal signature, ECM function, and cytoskeleton rearrangement that are induced by TGFβ (Figure 2B and Figure S4A-C). Gene expression scores of these pathways confirmed expression patterns caused by the treatment of cisplatin and by TGFβ (Figure 2B-F). Next, we conducted validation by RT-qPCR and the data confirmed the repression of epithelial marker genes and upregulation of mesenchymal signature genes by TGFβ, whereas such effect was overridden by cisplatin (Figure 2G-H). Similar changes of some of these genes were also revealed by Western blot analysis (Figure 2I). Similar trend of gene expression pattern was observed in MDA-MB-231 cells (Figure S4D-H). Altogether, our data indicate that EMT is a multi-step process that involves stepwise morphological and molecular changes, and the inhibition of cisplatin on cell migration occurs at early steps, i.e. cytoskeleton reorganization induced by TGFβ.

### Cisplatin suppresses cytoskeleton remodeling mediated by TGFβ through inactivation of TGFβ/SMAD signaling

**Figure 3:**
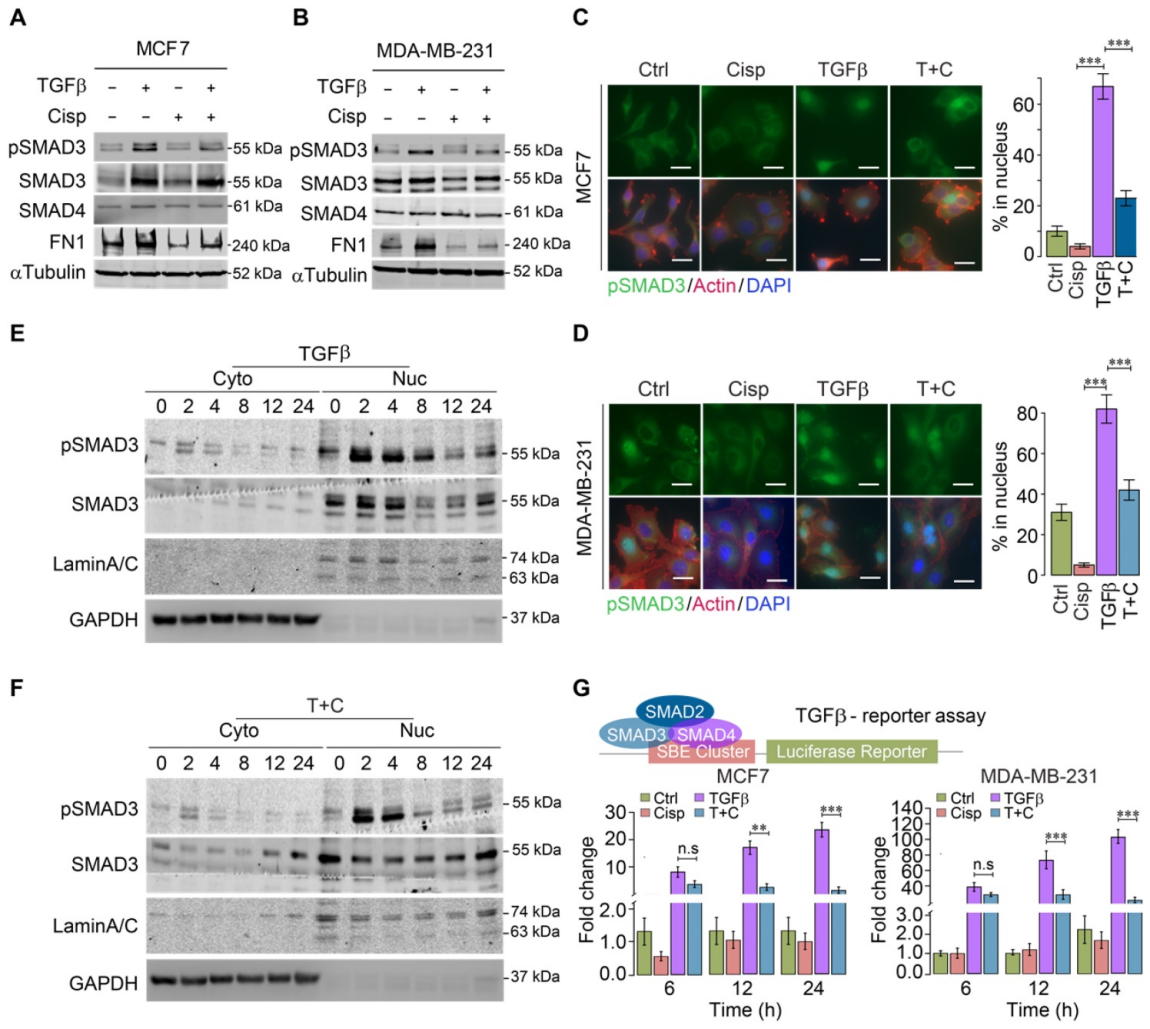
** Effect of cisplatin on TGFβ/SMAD3 signaling. A**,** B** Effect of cisplatin on pSMAD3 revealed by Western blot analysis for MDA-MB-231 (**A**) and MCF7 (**B**) cells. **C**,** D** Effect of cisplatin on nuclear pSMAD3 revealed by immunofluorescent staining of MDA-MB-231 (**C**) and MCF7 (**D**) cells. The quantification of ratio of pSMAD3 in nucleus were shown on the right panel. Bar = 50 μm. **E**,** F** Effect of cisplatin on TGFβ mediated SMAD3 phosphorylation (pSMAD3) revealed by Western blot of cell lysates from cytoplasm (**E**) and nucleus (**F**) during a time course from 0 to 24 h after cisplatin and/or TGFβ treatment. **G** Effect of cisplatin and/or TGFβ treatment of an SBE-luciferase reporter in MCF7 and MDA-MB-231 cells 48 h after the treatment. In (**C**,** D** and **G**), data represent means ± standard deviations (SDs). Concentration of drugs in this figure: TGFβ [5 ng/mL] cisplatin [10 μM]. Immunoblots shown in this figure have 3 replicates. ns, *, ** and *** means not significant, p < 0.05, p < 0.005, and p < 0.0005, respectively.

The strong influence of cisplatin on genes that are regulated by TGFβ suggests that cisplatin impairs TGFβ transcriptional activity. To provide evidence for this, we investigated whether cisplatin could block SMAD3 phosphorylation induced by TGFβ. Western blot analysis of MCF7 and MDA-MB-231 cells revealed that TGFβ treatment for 24 h significantly increased pSMAD3, which was repressed by cisplatin (Figure 3A-B). Once phosphorylated, pSMAD3 forms a complex with SMAD2 and SMAD4, and moves into the nucleus to execute the transcriptional activity of TGFβ signaling. Consistently, immunofluorescent staining of MCF7 and MDA-MB-231 cells using an anti-pSMAD3 antibody detected nuclear accumulation of pSMAD3 induced by TGFβ, which was abolished by cisplatin treatment (Figure 3C-D).

A time course study revealed that the levels of nuclear pSMAD3 were strongly increased 2 h after exposure to TGFβ, and significantly decreased at 8 h post-treatment. In the presence of cisplatin, pSMAD3 induced by TGFβ treatment at 2 h was not affected; however, pSMAD3 induction was gradually impaired starting from 4 h and displayed marked differences at 8 h after TGFβ treatment (Figure 3E-F).

It is known that phosphorylation of SMAD3 by TGFβ occurs through a phosphorylation cascade, i.e. TGFβ phosphorylates Type II receptor (TGFβ-RII), which recruits and phosphorylates Type I receptor (TGFβ-RI), leading to phosphorylation of SMAD3. The observation that inhibition of pSMAD3 by cisplatin occurs a few hours later than the initial phosphorylation of SMAD3 suggests that cisplatin does not directly inhibit kinase activity of this phosphorylation cascade; instead, it may affect transcriptional ability of TGFβ to activate its downstream target genes. To demonstrate this, we employed a TGFβ luciferase reporter (SBE-Luc), and detected a minor reduction of TGFβ transcriptional activity at 6 h after cisplatin treatment, whereas significant inhibition of TGFβ occurred at 12 and 24 h post-treatment (Figure 3G). These results collectively indicate that cisplatin counteracts effects of TGFβ signaling largely through suppressing transcriptional activity of TGFβ that plays a critical role in cytoskeleton remodeling.

### ATF3 stimulation by cisplatin suppresses FN1 transcription and hence compromises cell migration

To illustrate the molecular basis for this finding, we analyzed regulatory sequences of genes, whose expression is regulated by TGFβ, but counter regulated by cisplatin, i.e. genes involved in ECM, cytoskeleton, and EMT (Figure 2A-B). We found that activating transcription factor 3 (ATF3) binding site frequently appeared in the promoter, exon or intron of some genes, including fibronectin 1 (*FN1*), cytoskeleton organization and cell adhesion related genes, such as parvin beta (*PARVB*), serine/threonine-protein kinase (*PAK1*), integrins (*ITGB1*, *ITGB6*, *ITGA6* and *ITGA3*), latent-transforming growth factor beta binding protein 1 (*LTBP1*), myosin heavy chain 10 (MYH10), and *ATF3* itself (Figure S5A). ATF3 is a member of cAMP responsive element binding (CREB) family of transcription factor and can be induced upon physiological stress. Our analysis on the published ChIP data 39 indicated that ATF3 binds to the transcriptional start site (TSS) or body region of many genes, the binding of which is markedly increased by Camptothecin (CPT) treatment (Figure S5B). The analysis also revealed that ATF3 binds to several loci within the ATF3 gene and the binding is enhanced upon DNA damage, while the binding was absent in ATF3-KO cells (Figure S5C).

**Figure 4:**
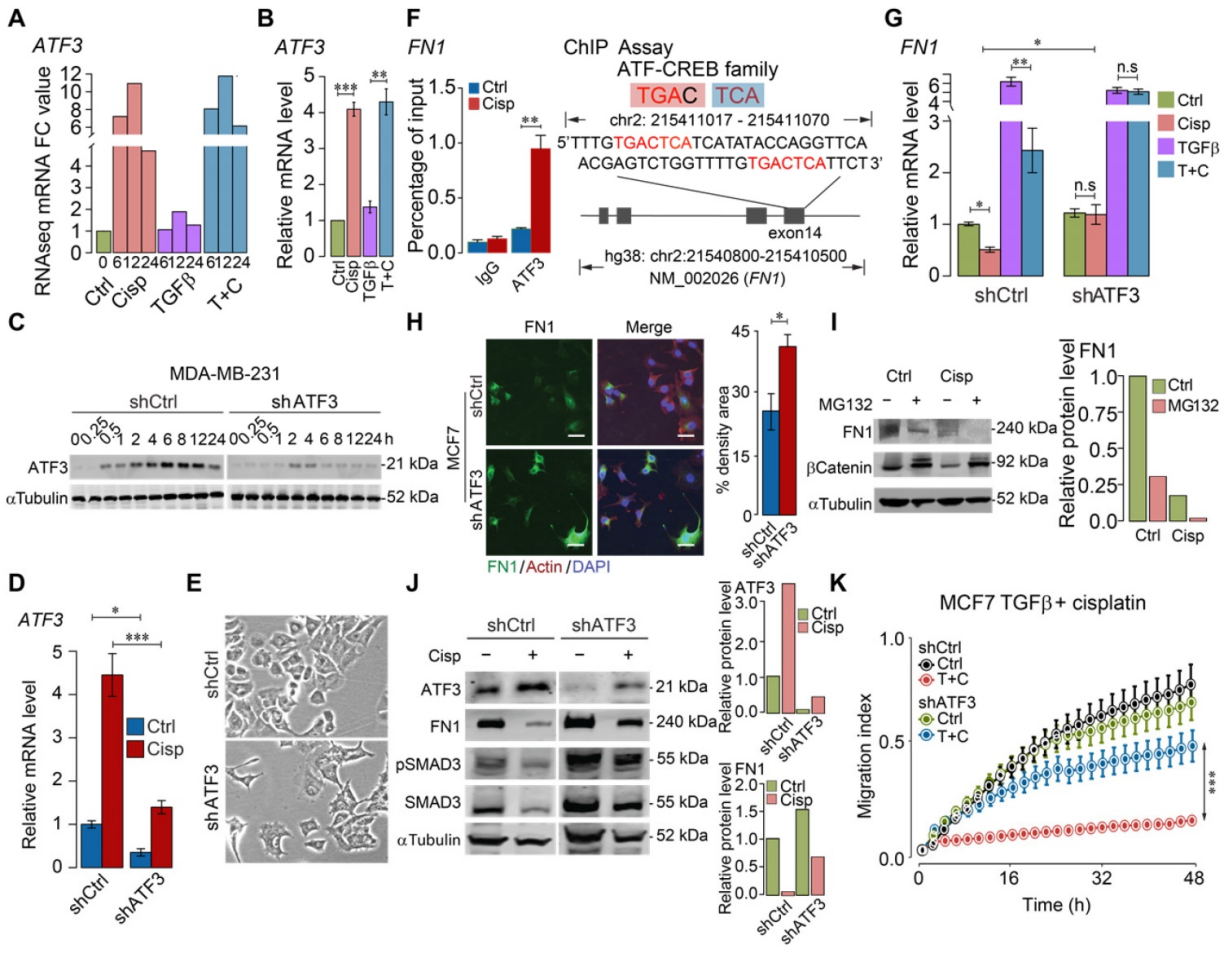
** ATF3 stimulation by cisplatin suppresses FN1 transcription and hence compromises cell migration. A**,** B** ATF3 expression was significantly induced by cisplatin but not affected by TGFβ revealed by RNA-seq (**A**) and confirmed by RT-qPCR (**B**) in MDA-MB-231 cells, FC (Fold Change). **C-E** Cisplatin induces ATF3 expression, which was diminished in ATF3-KD cells at protein (**C**) and RNA (**D**) levels. Also, ATF3-KD cells prevailed spindle cell phenotype (**E**). **F** ChIP-seq showed that ATF3 binds to exon 14 of *FN1*, which is enhanced by cisplatin treatment. The binding site was shown on the right panel. **G** Cisplatin reduces FN1 expression in both control and TGFβ treated cells, which is blocked by the knockdown of *ATF3*. **H** Knockdown of *ATF3* increased FN1 expression revealed in immunofluorescent staining of FN1 and merged with Actin and DAPI. Quantification of FN1 levels was shown on the right panel. Bar = 50 μm. **I** Western blot analysis of FN1 expression under ubiquitin-proteasome inhibitor, MG132, to determine if FN degradation requires proteasome-mediated protein degradation in control cells and cisplatin treated cells. **J** Cisplatin induces ATF3, reduces FN1 and pSMAD3, which is attenuated by knockdown of ATF3. Quantification of ATF3 and FN1 levels was shown on the right panels. **K** *ATF3* knockdown did not affect cell migration but significantly attenuated inhibition of cisplatin on cell migration induced by TGFβ treatment. In (**B**, **D**, **F**, **G**, **H** and **K**), data represent means ± standard deviations (SDs). See also Figure S5. Concentration of drugs in this figure: TGFβ [5 ng/mL] cisplatin [10 μM]. Immunoblots shown in this figure have 3 replicates. ns, *, ** and *** means not significant, p < 0.05, p < 0.005, and p < 0.0005, respectively.

Previous studies showed that cisplatin could induce expression of ATF3, yet the significance of such induction remains elusive 40, 41. To investigate whether cisplatin affects expression of these genes through ATF3, we first examined RNA-seq data for ATF3 expression and found that ATF3 was upregulated by cisplatin as early as 6 h post-treatment, and such induction was not affected by TGFβ (Figure 4A). The upregulation of ATF3 upon cisplatin treatment, but not by TGFβ, was confirmed by RT-qPCR (Figure 4B). To further study the function of ATF3, we knocked down *ATF3* (Figure 4C-D) and found that *ATF3* knockdown (ATF3-KD) cells prevailed spindle cell phenotype reminiscent of EMT (Figure 4E).

Next, we examined if ATF3 could bind to FN1, which is induced by TGFβ, and bears an ATF binding site in exon 14 of the gene. FN1 is a constitutive extracellular matrix protein that plays important role in cell adhesion, cell motility and wound healing 42. Our ChIP-PCR analysis revealed that ATF3 indeed binds to this site and the binding was enhanced upon cisplatin treatment (Figure 4F). RT-qPCR analysis indicated that TGFβ could enhance transcription of FN1, which is partially inhibited by cisplatin, and such inhibition is reversed when *ATF3* was knocked down (Figure 4G). *ATF3* knockdown also increased FN1 protein level revealed by immunofluorescent imaging (Figure 4H). We further showed that the inhibition of cisplatin to FN1 protein level was not affected by MG132, a potent proteasome inhibitor (Figure 4I), which is consistent with our observation that cisplatin affects FN1 on the transcriptional level.

After confirming that cisplatin induces expression of ATF3, next, we investigated if knockdown of *ATF3* could antagonize the effect of cisplatin on TGFβ. Our data indicated that knockdown of *ATF3* attenuated the inhibition effect of cisplatin on FN1 and pSMAD3 (Figure 4J). Our further analysis showed that shATF3 alone had no obvious effect on cell migration (Figure 4K, upper two curves), however, it significantly attenuated the inhibitory effect of cisplatin on cell migration induced by TGFβ (Figure 4K, lower two curves), supporting the notion that ATF3 mediates the antagonizing effect of cisplatin on TGFβ signaling.

**Figure 5:**
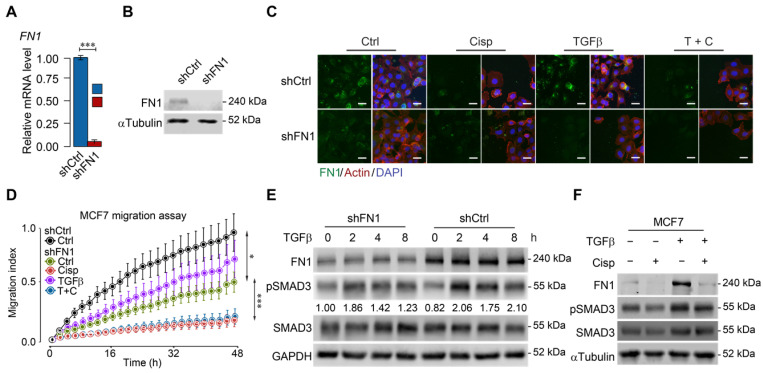
** ATF3 stimulation by cisplatin suppresses FN1 transcription and hence compromises cell migration. A**,** B** Confirmation of *FN1* knockdown by shRNA using RT-qPCR (**A**) and Western blot (**B**) in MCF7 cells. **C** Expression and distribution of FN1 under TGFβ, cisplatin or combined treatment showed by immunofluorescent staining. *FN1* knockdown cells mimic the morphology of cisplatin-treated MCF7 cells. Bar = 50 μm. **D** *FN1* knockdown inhibits migration of MCF7 cells under cisplatin addition to TGFβ treatment. **E** *FN1* knockdown reduces SMAD3 phosphorylation in both conditions with/without TGFβ-treatment by Western blot. The ratios of pSMAD3/SMAD3 are shown under the band of pSMAD3. **F** TGFβ induces expression of FN1 and pSMAD3 in MCF7 cells, which is inhibited by cisplatin treatment. In (**A** and** D**), data represent means ± standard deviations (SDs). Concentration of drugs in this figure: TGFβ [5 ng/mL] cisplatin [10 μM]. Immunoblots shown in this figure have 3 replicates. ns, *, ** and *** means not significant, p < 0.05, p < 0.005, and p < 0.0005, respectively.

To further study the relationship among cisplatin, ATF3 and TGFβ/SMAD signaling, we knocked down *FN1* (Figure 5A-B) and found that FN1-KD cells mimicked the morphology of cisplatin-treated cells (Figure 5C). Furthermore, cell migration assay demonstrated that knockdown of *FN1* not only markedly retarded cell migration but also attenuated the stimulatory effect of TGFβ (Figure 5D). Consistent with these observations, Western blot analysis revealed that TGFβ induced FN1 at a time course that was accompanied by increased pSMAD3, whereas *FN1* knockdown significantly decreased the basal level of pSMAD3 (Figure 5E). Meanwhile, treatment of cisplatin antagonized the induction of TGFβ to FN1 and pSMAD3 (Figure 5F). These data are consistent with a model through which cisplatin stimulates ATF3 expression that suppresses TGFβ and then FN1, hence compromising EMT and cell migration. However, to our surprise, Western blot analysis revealed that *FN1* knockdown not only decreased the basal level of pSMAD3, but also inhibited TGFβ-induced SMAD3 phosphorylation (Figure 5E). These data suggest that FN1, while being positively regulated by TGFβ, also plays a role in maintaining TGFβ activity.

Altogether, these data indicated that cisplatin activates ATF3, which, upon induction, activates its own expression and represses a positive loop constituted among FN1 and TGFβ, leading to impaired activity of TGFβ signaling, which compromises EMT and cell migration.

### Cisplatin blocks cancer metastasis and inhibits breast cancer growth together with paclitaxel in neoadjuvant chemotherapy

Given that cisplatin could inhibit EMT and cell migration through inhibiting the activity of the genes involved in this process, we wanted to explore the potential of our finding *in vivo* by using a highly metastatic 4T1 cell line, which was derived from a mammary tumor of BALB/c mice and, therefore, could be implanted into the mammary fat pad of BALB/c mice without suffering from immune rejection. 10 days after the implantation of 1×10^6^ GFP-labeled cells into the mammary fat pad of BALB/c mice, tumors reached about 0.5 cm in diameter on average. We treated the host mice with a dose of cisplatin at 6 mg/kg 3 times with each treatment 3 days apart. 9 days following the last treatment, we removed the tumors and followed the mice for another 7 days before sacrificing them for examining lung metastasis (Figure S6A). Our data indicated that cisplatin treatment could effectively inhibit cancer growth, as reflected by significantly reduced tumor size and volume, and diminished cancer metastasis, as reflected by lung colonization (Figure S6B-F). However, our data also indicated that although effective, cisplatin at this dose was quite toxic, as reflected by body weight loss of the recipient animals (Figure S6G).

**Figure 6:**
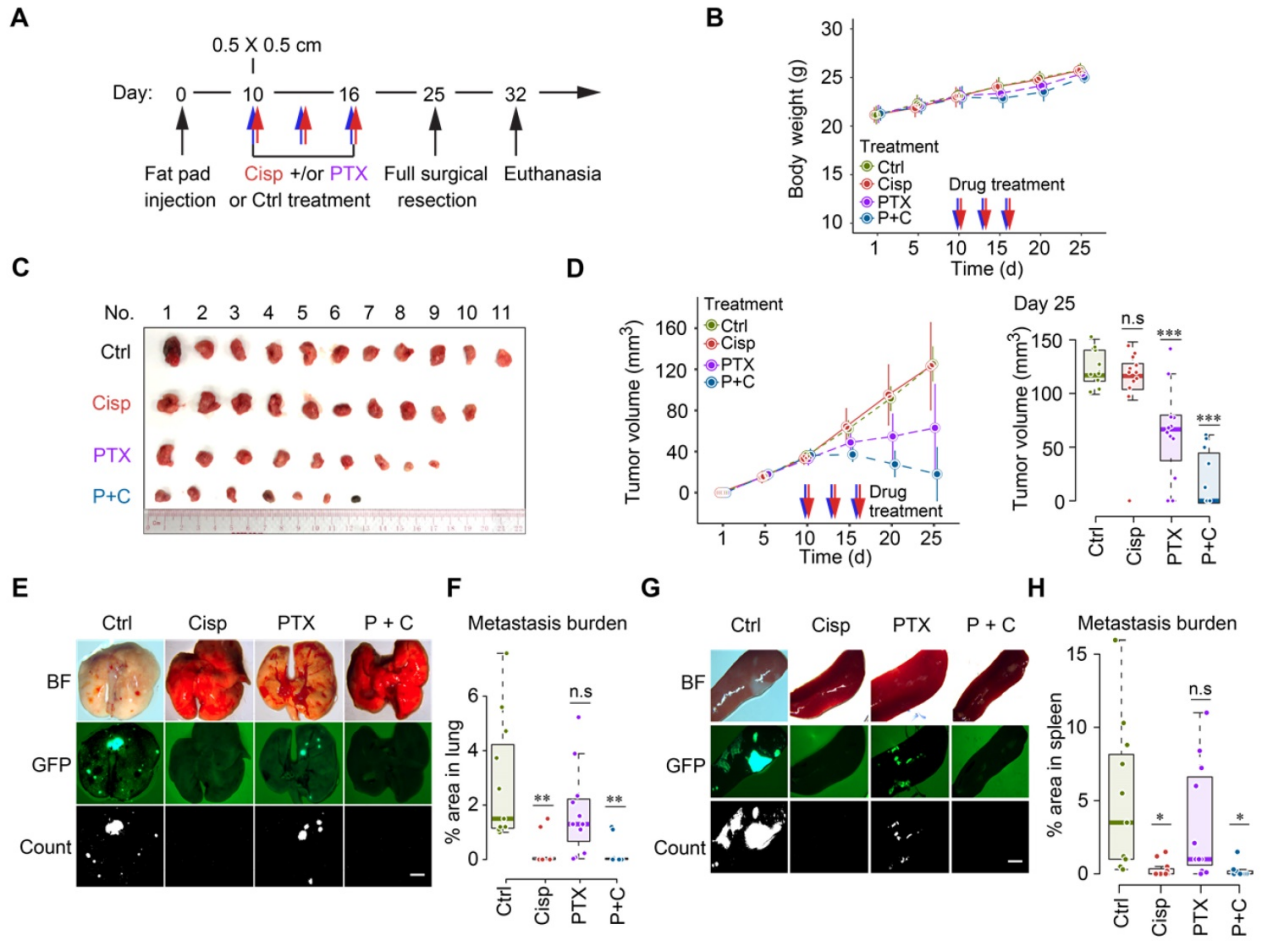
** Cisplatin blocks cancer metastasis in a neoadjuvant setting in nude mice. A** A procedure of allograft of 4T1 cells by implanting them into the mammary fat pad of BALB/c mice followed by treatment with low dosage of cisplatin [2 mg/kg] and/or paclitaxel [18 mg/kg] at the time point indicated. **B** Body weights of control and cisplatin treated mice during the 25 days experiment period. **C**,** D** Volumes, and sizes of tumors during 25 days of the treatment with cisplatin and/or paclitaxel. Quantification of tumor volumes of different groups in day 25 were shown on the right panels (**D**). **E-H** Measurement of metastasis of tumors to lungs (**E**, **F**) and spleens (**G**, **H)** at 25 days under different treatment conditions as indicated, BF (Bright Field). GFP intensities were measured and quantified in (**F**, **H)**. * indicates p < 0.05 and ** indicates p < 0.01 in relation to controls. Bar = 3 mm. In (**B**, **D**, **F** and **H**), data represent means ± standard deviations (SDs). See also Figure S6. ns, *, ** and *** means not significant, p < 0.05, p < 0.005, and p < 0.0005, respectively.

Our earlier data indicated that lower doses of cisplatin could effectively block movement of cultured cells as indicated by greatly reduced filopodium formation 34, therefore we empirically reduced doses of cisplatin to study their effect on cancer growth and metastasis following the same treatment protocol (Figure S6A). We found that mono-treatment of cisplatin at 2 mg/kg did not have a significant effect on body weight and tumor growth (Figure 6A-D), but blocked cancer metastasis to lungs and spleens (Figure 6E-H). This finding indicates that this dose of cisplatin effectively reduced toxicity and blocked tumor metastasis, while it does not inhibit primary tumor growth, highlighting the necessity for combination therapy like other studies showed 43-45.

Recent studies showed that paclitaxel, despite its effect in inhibiting tumor growth, could cause cancer metastasis in a neoadjuvant setting 46-48. We then tested the combination of low dose cisplatin and paclitaxel for the neoadjuvant chemotherapy of metastatic breast cancer. Thus, we specifically tested potential effects of mono- and combined treatment of paclitaxel (18 mg/kg, a regular dose for mice) together with cisplatin (2 mg/kg) on toxicity, tumor growth and metastasis (Figure 6A). Our data revealed unapparent toxicity under these treatment conditions as measured by body weight (Figure 6B). Mono-treatment of paclitaxel significantly reduced tumor growth compared with control and mono-treatment of cisplatin (Figure 6C-D). Of note, cisplatin and paclitaxel combined treatment was most effective in preventing tumor growth, indicating an improved effect of the combination (Figure 6C-D). The most striking difference lies in metastasis: while control mice showed extensive tumor foci on the lungs (Figure 6E-F) and spleens (Figure 6G-H), paclitaxel mono-treated mice had fewer, yet statistically insignificant, number of foci on these organs. In contrast, no obvious foci were detected in cisplatin or cisplatin/paclitaxel combined treated mice. Hence, we concluded that regular dose of paclitaxel combined with a lower dose of cisplatin could be successful as a neoadjuvant regime not only for reducing tumor size but also for inhibiting cancer metastasis in engrafted mammary tumor mouse model.

### Cisplatin inhibits actin cytoskeleton rearrangement and blocks lung metastatic colonization

**Figure 7:**
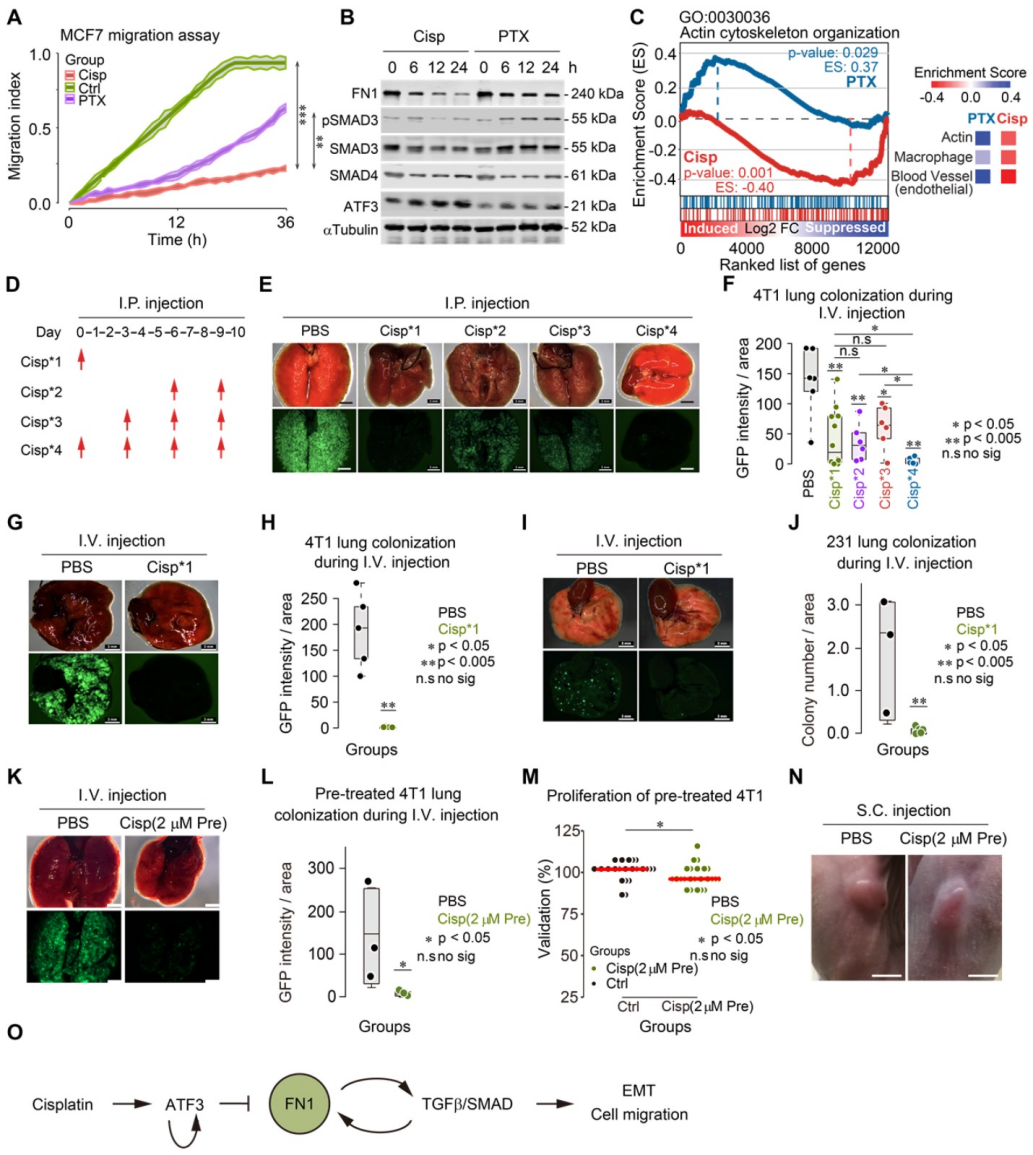
** Cisplatin blocks cancer metastasis through inhibiting early colonization. A** The effects of cisplatin and paclitaxel on MCF7 cell migration were compared with control group. **B** Western blot shows the effects of cisplatin and paclitaxel on expression of FN1, ATF3, SMAD3, SMAD4 and phosphorylation of SMAD3 in a 24-h time lapse. Concentration of drugs: Cisplatin [10 μM] PTX [5 μM]. Replicate = 3. **C** GSEA enrichment score of actin cytoskeleton organization (GO: 0030036) are different under cisplatin and paclitaxel treatments. Comparison of enrichment scores of actin, macrophage, and blood vessel under two treatments were shown on the right panel. **D** A procedure of tail vein injection (i.v.) of pre-trained tumor cells and intraperitoneal injection (i.p.) of cisplatin. Pre-trained breast tumor cells were injected through i.v. into each mouse at day 0. Then, cisplatin or PBS were injected through i.p. depending on the designing of each group. All the mice were killed at day 10. **E**, **G** and** I** Images of colonized lungs by pre-trained 4T1 cells and pre-trained MDA-MB-231 cells (these cells have lower colonization ability compared with pre-trained 4T1 cells) after cisplatin treatments by dissection. The upper panel are the bright viewed images of colonized lungs while the lower panel are the immunofluorescent images showing GFP signals. Bar = 3 mm. **F** Summary and comparison of GFP intensity/area of colonized lungs by pre-trained 4T1 cells. The intensity of GFP and the area of each lobby of all groups were analyzed by ImageJ. **H**,** J** Summary, and comparison of GFP intensity (or number of colonization)/areas of colonized lungs by pre-trained 4T1/pre-trained MDA-MB-231 cells in PBS and cisplatin injected (only at first time through i.v.) groups. **K** Images of colonized lungs by pre-trained 4T1 cells with/without cisplatin [2 μM] pre-treatment by dissection. Bar = 3 mm. **L** Summary and comparison of number of GFP puncta of colonized lungs by pre-trained 4T1 cells with/without cisplatin [2 μM] pre-treatment (i.v.) groups. **M** Comparison of effects of PBS and cisplatin [2 μM] pre-treatment on proliferation. **N** Images of tumors formed by PBS and cisplatin pre-treated 4T1 cells by subcutaneous (s.c.) injection. Bar = 5 mm. **O** Summary of the mechanism of cisplatin inhibition on cell migration/cancer metastasis through ATF3, which regulates the positive reciprocal loop among FN1, TGFβ/SMAD. In (**A**,** F**,** H**,** J**,** L** and **M**), data represent means ± standard deviations (SDs). ns, *, ** and *** means not significant, p < 0.05, p < 0.005, and p < 0.0005, respectively.

To understand the differential response to cisplatin and paclitaxel treatment in cancer metastasis, we studied cell migration and gene expression in cultured 4T1 cells upon treatment. Consistent with our observation on cancer metastasis *in vivo*, our *in vitro* data revealed a less potent inhibition of cell migration by paclitaxel compared to cisplatin (Figure 7A). Of note, while cisplatin induced ATF3, which inhibited expression of FN1 and pSMAD3, paclitaxel did not affect expression of ATF3, although slightly increased pSMAD3 and reduced FN1 were observed (Figure 7B). Gene expression analysis also revealed that cisplatin repressed expression of genes involved in actin cytoskeleton arrangement, whereas paclitaxel induced some of those genes in MDA-MB-231 cell line (Figure 7C). Similar change of markers was also detected in the implanted tumors in mice after treatment by immunohistochemistry (IHC) (Figure S6H).

Cancer metastasis is a complex process and involves many steps 2, 4, 11, 49. Because cisplatin inhibited cancer metastasis and actin cytoskeleton rearrangement, we hypothesized that cisplatin might inhibit cell colonization on target organs/tissues, which is one of the early steps for cancer metastasis. To test this, we employed a pre-trained 4T1 cell model, which could form lung metastatic colonization within 10 days after tail vein injection (i.v.).

We followed a procedure to inject 5×10^5^ GFP^+^ 4T1 cells through the tail vein of BALB/c mice and monitored lung colonization after injection of cisplatin (intraperitoneal, i.p.) at the different time points as indicated (Figure 7D). In the control group (PBS injection only), cancer cells fully colonized the lungs 10 days after their inoculation (Figure 7E-F). In contrast, mice receiving cisplatin at all 4 time points (Cisp*4) were free of cancer colony on the lungs, suggesting that cisplatin effectively blocked cancer metastasis (Figure 7D-F). However, the failure of lung metastasis due to cisplatin treatment might result from either the inhibition of initial colonization or the growth of cancer cells on the lungs. To distinguish this, we compared the effect of cisplatin treatment at the 1^st^ time point-only (Cisp*1) with those treated at the last 2 time points (Cisp*2) and at the last 3 time points (Cisp*3). The data indicated that cisplatin treatment at later time points (Cisp*2 and Cisp*3) partially inhibited lung metastasis with reduced GFP foci on the lungs of all recipient mice (Figure 7E-F), whereas Cisp*1 completely blocked metastasis in 50% of mice while the other 50% of mice exhibited lower GFP signal (Figure 7E-F). We believed that the variation of Cisp*1 might be caused by several factors: 1) Individual difference of recipient mice; 2) Cisplatin administered via i.p. might take time to gradually diffuse into the blood and reach the lung, while cancer cells administered via i.v. could reach the lung immediately after injection, which might lead to variable effects. To investigate this, we injected cisplatin through i.v. at the same time when cancer cells were injected to avoid the delay, the data indicated that the concurrent administration of cisplatin and cancer cells could completely blocked lung colonization of the cancer cells (Figure 7G-H). Similar data was also obtained when pre-trained MDA-MB-231 cells were used for lung colonization experiment (Figure 7I-J).

We showed earlier that cisplatin blocks cytoskeleton rearrangement, which is required for the early steps of EMT. We hypothesized that this effect of cisplatin might play an important role in cancer metastasis. To testify this hypothesis, we pre-treated 4T1 cells with a lower dose of cisplatin (5 μM, or 1.5 mg/L, which should be lower than 2 mg/kg used for animal treatment) for 6 h followed by washing the cells with PBS and injected them through i.v. into BALB/c mice. We found that the pre-treatment of cisplatin completely blocked lung colonization, while slightly inhibit proliferation and tumorigenesis (Figure S6I-K). So, we further reduced the concentration of cisplatin to 2 μM (0.6 mg/L) and performed the pre-treatment and injection as above. We found that the pre-treatment of 2 μM cisplatin also blocked lung colonization (Figure 7K-L) and had little effect on proliferation and tumorigenesis (Figure 7M-N).

These data demonstrated that the lower dose of cisplatin could inhibit early steps of EMT and block lung metastatic colonization while having little effect on proliferation and tumor growth.

### Clinical evidence for potential prognostic cancer metastasis biomarkers

To test whether the inhibitory effect of cisplatin on metastasis was due to the inhibition of tumor microenvironment of metastasis (TMEM) consisting of direct contact between cancer cells and macrophages, endothelial cells, *etc*, in combination with actin cytoskeleton rearrangement, we compared gene set enrichment analysis (GSEA) of tumors from mice upon paclitaxel and cisplatin treatments, respectively. From the enrichment analysis, besides expression of genes involved in actin cytoskeleton organization, macrophage, endothelial cell (Figure 7C), cisplatin also downregulates expression of genes related to ECM organization, filopodium, and vascular endothelial growth factor receptor (VEGF) signaling pathway in cisplatin treated breast cancer cells, as compared to paclitaxel treatment (Figure S7A).

To verify the clinical significance of these genes in human patients, we analyzed the relationship between these genes' expression and survival rates of human patients from The Cancer Genome Atlas (TCGA) database. Higher mRNA expression was detected in *FN1* and *LTBP1* in breast cancers than normal population, with increasing expression levels from primary to metastatic cancers, whereas *ATF3* had reversed expression patterns (Figure S7B). Consistent with these expression patterns, high levels of *FN1* and *LTBP1* as well as low level of *ATF3* were correlated with lower overall survival (OS) rate (Figure S7C).

To understand the effect of these genes on metastasis in clinic, we further analyzed the distant metastasis free survival (DMFS) rate in patients' therapies. In patients with distant metastasis, the expression levels of *FN1* and *LTBP1* were higher than distant metastasis free patients (Figure S7D-E), while the expression level of *ATF3* was lower than distant metastasis free patients (Figure S7F). From these metastasis-associated animal and clinical data, it was verified that high expression level of *ATF3* was correlated with inhibited metastasis or colonization, while high expression levels of *FN1* and *LTBP1* were correlated with enhanced metastasis or colonization. Hence, targeting on effector genes provided better outcome than targeting on transcriptional modulators.

## Discussion

Cisplatin resistance frequently occurs during cancer therapy 26-28, 30, 50-52. In a *Brca1* mutant mouse model, we have previously shown that prolonged treatment of cisplatin induced cisplatin resistance. However, despite the failure of blocking tumor growth, cisplatin elicited a strong effect in suppressing tumor metastasis 34, 44, 45, 53, 54. This finding suggests that the effect of cisplatin on cancer growth and metastasis might involve distinct mechanism. Consistent with this notion, this study exemplified that a lower dose of cisplatin only inhibited cancer metastasis without an obvious effect on tumor growth. For the underlying mechanism, our analysis revealed that cisplatin inhibited a positive reciprocal regulation loop formed among FN1 and TGFβ that is essential for activation of TGFβ, and such an effect was abolished after disruption of ATF3.

### ATF3 plays a critical role in mediating inhibitory effect of cisplatin on TGFβ/SMAD signaling

Previous studies indicated that ATF3 can be induced by several factors, including p53, JNK, and cisplatin, playing a dual role in cancer growth and metastasis 40, 55-57. Our data indicated that cisplatin induced expression of many genes involved in cell cycle, apoptosis, DNA damage response and repair (Figure 2A). Because cisplatin treatment quickly induces DNA damage, as evidenced by accumulation of nuclear γH2AX at approximately 1-2 h after cisplatin addition 53, we believed that the induction of these genes by cisplatin may be primarily triggered by DNA damage responses. It is well known that host cells, after detecting DNA damage, could hold cell cycle progression and initiate DNA damage repair by triggering expression of relevant genes 58-62.

Some previous studies mentioned that ATF3 promotes activation of matrix metalloproteinase 13 and differentiation related genes after being stimulated by TGFβ signaling, which is mediated by interacting with SMAD proteins 63, 64. Some other studies also found that ATF3 suppressed TGFβ signaling through interacting with SMAD proteins 65 or inhibiting phosphorylation of p38 66. Conversely, there was a report showing that ATF3 enhances TGFβ signaling in malignant derivative of breast cancer cells by long time or stable overexpressed/knocked down cells, the condition of which is significantly different from ours 67.

Our data indicated that* ATF3* was strongly induced by cisplatin hours after the treatment among genes induced by cisplatin upon DNA damage. Interestingly, upon the induction by cisplatin, *ATF3* binds to its regulatory region to sustain its own expression (Figure S5C). Because knockdown of *ATF3* abolished inhibitory effects of cisplatin on TGFβ-mediated transcription, EMT, and cell migration, we concluded that ATF3 mediates the inhibitory action of cisplatin and plays a pivotal role in repressing cell migration, EMT and metastasis induced by TGFβ/SMAD signaling.

### FN1 and TGFβ constitute a positive reciprocal regulation loop that mediates functions of TGFβ signaling in early steps of EMT

TGFβ is produced in the cytoplasm, and forms a homodimer, which interacts with a latency associated peptide (LAP) to form a complex called small latent complex (SLC) 68. SLC remains in the cytoplasm until it is bound by LTBP1, forming a larger complex called large latent complex (LLC), which is secreted to the ECM 69. It was shown that FN1 interacts with LTBP1 in the ECM, which is required for latent TGFβ activation 70, 71. While our data confirmed this positive regulation of TGFβ activation by FN1, we found that TGFβ also positively regulates FN1 transcription, thus these two genes constitute a positive reciprocal regulation loop to maintain their activities. *FN1* contains an ATF3 binding motif in its regulatory region and the binding of ATF3 represses its expression. Because ATF3 is strongly induced by cisplatin, and ATF3 affects several processes through interacting with SMAD proteins in TGFβ pathway 63, 64, this data provides a molecular basis accounting for the reason why cisplatin elicits a strong effect in antagonizing TGFβ mediated transcriptional activity (Figure 2).

While the length of EMT induction *in vivo* cannot be easily defined, it is a common practice to induce EMT *in vitro* under defined conditions. We have been using TGFβ to induce EMT and found it is indeed mediated by SMADs, as this process could be blocked by the disruption of SMAD4 72, which constitutes the SMAD2/3/4 complex for executing its function 21, 22. Our current study has also conferred to a deeper understanding of this issue. First, we found that molecular actions required for EMT occur quickly (i.e. a few hours after cisplatin treatment) before the onset of the morphological transition towards EMT; and cisplatin treatment for 6 h impairs expression of a variety of genes involved in the cytoskeleton rearrangement and ECM formation, leading to the blockage of EMT. These findings not only provide strong evidence for the multi-step induction of EMT, but also reveal the crucial role of cytoskeleton and ECM in the early steps of EMT. Two recently published studies indicated that EMT is dispensable for metastasis when tested in two animal models 9, 10, whereas a more recent study revealed that cells at early stage of EMT, while maintaining their epithelial status, are a major source of metastasis 8. Our data indicates that cells at different stages of EMT still maintain their ability to migrate and cispatin, which blocks the earliest changes of EMT, can block cell migration *in vitro* and cancer metastasis *in vivo*.

### Cisplatin effectively blocks breast cancer metastasis and inhibits cancer growth together with paclitaxel in neoadjuvant chemotherapy

Paclitaxel is a therapeutic drug for several types of solid cancers, including breast, ovarian, lung, bladder, prostate, melanoma, and esophageal cancers. Although, some studies revealed the effect of paclitaxel on inhibiting EMT 73, 74, it is most commonly used for neoadjuvant treatment of breast cancer 48, 75, 76. Of note, several recent studies indicated that paclitaxel, when used as a neoadjuvant agent in animal models, although delayed tumor growth, induced cancer metastasis through various mechanisms 46, 48, 77-80. In our neoadjuvant chemotherapy study, we found that paclitaxel delayed tumor growth, but did not induce tumor metastasis. Instead, a slight reduction of tumor metastasis was observed. This difference might be caused by the fact that our study used 4T1 cells that were pre-trained for high frequency lung metastasis (100% recipient mice developed lung metastasis after removal of primary tumors in 10 days), whereas cells with less defined metastatic ability were used in other studies. Thus, our finding, which is consistent with the potency of paclitaxel on breast cancer, suggests that the recent findings on the role of paclitaxel in inducing cancer metastasis might only occur in some specific conditions.

Continuing on this line, our study has made two noticeable findings: 1) A lower dose of cisplatin (2 mg/kg) blocks cancer metastasis without apparent side effects. We further showed that pre-treatment of cisplatin at a lower dose for 6 h *in vitro* could block lung metastasis, but not the growth of these cells on the primary injection site. These observations suggest that the mechanisms underlying the effect of cisplatin on cancer metastasis and growth are distinct. 2) The lower dose of cisplatin alone was insufficient to inhibit tumor growth, yet it elicited a significant effect on retarding tumor growth and blocking tumor metastasis in a neoadjuvant setting. Because both paclitaxel and cisplatin are commonly used for therapeutic treatment of many different types of human cancers 26, 48, 75, 76, 81, our finding should have widespread potential for chemotherapy application.

In summary, our study has made several findings: 1) Cisplatin induces transcription regulation factor ATF3 expression, which suppresses a variety of cytoskeleton, ECM, filopodia and adhesion related genes; 2) Suppression of transcription of *FN1* by ATF3 compromises EMT and cell migration; 3) TGFβ also induces FN1 to form a positive feedback loop to maintain its expression; and 4) Cisplatin and paclitaxel, in a neoadjuvant chemotherapy setting, block cancer metastasis together by inhibiting colonization of cancer cells on the target organs and cancer growth. Based on these findings, we proposed a model that cisplatin activates ATF3, which represses the positive reciprocal regulation loop formed between FN1 and TGFβ. This action, consequently, blocks the early step in the EMT; compromises cell migration *in vitro*; and cancer metastasis *in vivo* (Figure 7O).

## Methods

### Cell culture

All cells were cultured on monolayer with Dulbecco's Modified Eagle Medium (DMEM) or RPMI 1640 medium supplemented with 10% fetal bovine serum (FBS) or 2-10% charcoal-stripped serum (Gibco), 100 U/mL Penicillin-Streptomycin and 2 mM L-glutamine. Cells were incubated at 37 °C in a 5% CO_2_ humidified incubator and passaged every 3-4 days. All cell lines were tested and free for mycoplasma contamination.

### Lentivirus infection

HEK293T cells were thawed from liquid nitrogen and cultured in DMEM containing 10% FBS, growing and passage at least three times. Lentiviruses were produced by transfecting the HEK293T cells with knocking down plasmid shFN1 or shATF3, and the psPAX2 (Addgene plasmid #12260) and pMD2.G (Addgene plasmid #12259). The transfections were carried out using the Lipofectamine 2000 (Invitrogen) according to the manufacturer's instructions. The virus-containing medium was harvested 48 or 72 h after transfection and subsequently pre-cleaned with a 3,000×g centrifuge and a 0.45 μm filtration (Millipore). The viruses were used for titration and infection freshly or stored at -80 °C freezer.

### Cell migration assay

Cell migration was monitored by measuring impedance of cells migrating across Boyden chambers on a Roche xCELLigence RTCA (Real-Time Cell Analysis) Analyzer 37 in a 37 °C incubator supplemented with 5% CO_2_. Cells were seeded on CIM-16 plates (for detecting Cell Invasion and Migration) in duplicate wells and subject to analysis following manufacturer's protocol. Migration index was calculated by RTCA Data Analysis Software automatically after the measurement.

### Filopodium index

Choose 3-5 fields of each repeat (well or dish) and count the number of all the cells in each field. Count the number of filopodium of each cell. Calculate the average number of filopodium per cell of each repeat and group.

### Luciferase activity assay

pGL3B vector containing different promoter sequences were transfected into MDA-MB-231, MCF7, mouse 69 cells. All transfections were performed with LipofectamineTM 2000 (Invitrogen). After a 24-h incubation, those cells were treated by different conditions at different time point. At the end point, luciferase activity was assessed with the Dual-Luciferase Reporter Assay Kit (Promega). The SMAD/SEB-Luc was obtained as a gift from Prof. Xinhua Feng 82.

### Western blot

Different types of cells were initially treated with 0.1% DMSO in the absence and presence of 10 μM cisplatin/5 ng/mL TGFβ, on a 6-well plate at 37 °C in a 5% CO_2_ humidified incubator for the indicated time on each well. Cells were then washed with PBS thrice. Subsequently, gel loading dye was directly applied to the adherent cells, incubated at room temperature for 10 min, and transferred to heat-block for protein denaturation at 95 °C for 10 min. Protein samples were separated on 10% or 15% SDS-PAGE (sodium dodecyl sulphate-polyacrylamide gel electrophoresis) followed by Western blot using specific antibodies (Table S1). Blots were scanned by near-infrared fluorescence using Licor Odyssey CLx Imager. Quantifications of the Western blot were performed using Image J.

### Immunofluorescent staining

Monolayer cultured cells were washed with PBS thrice. Subsequently, cells were fixed with 10% Formalin and washed with 1% Triton X-100, and then stained with correspondent antibodies staining by using methods described previously 34. 10× to 40× magnified images were taken using Olympus BX83 Upright Fluorescent Microscope, whereas 63x magnified images were taken on Carl Zeiss LSM 710 Confocal Fluorescent Microscope, and then fluorescent signal intensity and localization of signals was analyzed by ImageJ. Antibodies for immunofluorescent staining are listed on Table S1. Filamentous actin was stained by 1:1000 phalloidin.

### Immunohistochemistry (IHC)

Resected tumors and organs were fixed in 10% Formalin, then dehydrated by xylene and embedded in paraffin. The blocks were cut into 5-10 μm slices, then the slices were transferred to 25×75 mm positive adhesion glass slides and hydrated by ethanol, followed by IHC staining following manufacturer's instructions for Thermo Fisher Histostain-*Plus* IHC Kit. IHC slides were counterstained by Hematoxylin. All slides were dehydrated by xylene and mounted by DPX Mountant. 10× magnified images were taken using Olympus BX83 Upright Fluorescent Microscope. Antibodies for IHC are listed on Table S1.

### Quantitative real-time PCR

To monitor and validate the candidate genes. Total RNA was isolated with TRIzol reagent (Thermo Fisher Scientific, Carlsbad, CA) according to the manufacturer's instructions. Reverse transcription was performed using QuantiTect Reverse Transcription Kit (QIAGEN). Real-time PCR reactions were performed using FastStart Universal SYBR Green Master (Roche, 4913850001) on QuantStudio™ 7 Flex Real-Time PCR System (Thermo Fisher Scientific). Relative quantification was achieved by normalization to the amount of 18S. Primers used for real-time PCR are listed on Table S2.

### qChIP assay

Cisplatin treated or untreated cells were cross-linked with 1% formalin for 15 min and ChIP was performed with ATF3 antibody by using methods described previously 83. Primer sequences are listed on Table S2.

### Allograft and xenograft mouse models

All experiments were approved by University of Macau's Animal Care Ethics Committee and adhere to the guidelines of the Macau's Council on Animal care. All animal operation was strictly procedure in accordance with approving from Animal care and Use committee of the Faculty of Health, University of Macau. Briefly, 6-10 weeks old female into wild type or athymic nude mice were implanted with 4T1 mouse cancer cells in the bilateral 4^th^ mammary fat pads. For all xenograft studies, mice were randomly assigned to experimental group. 1×10^6^ cells were orthotopic implanted, and tumors became visible 7-14 days post-implantation. Paclitaxel and cisplatin treatment were initiated when tumors reached 0.5 cm in diameter on average. Mice were intraperitoneally injected with cisplatin at doses of 2 or 6 mg/kg body weight 3 times (each time 3 days) with or without paclitaxel treatment at 18 mg/mL continuously. Tumor volume was measured 2-4 times per week and compared between different treatment groups of mice (N = 9-12 tumors per group). Tumor volume was calculated using the formula: *V* = *ab*^2^/2, where a and b is tumor length and width, respectively. All the mice were sacrificed in the experiments.

### RNA-Seq data processing

Total RNA from treated and untreated cells at each time point (0, 6, 12 and 24 h) were processed using RNeasy mini kit (QIAGEN), and RNA concentration and integrity were measured using the Agilent 2100 Bioanalyzer (Agilent Technologies). cDNA libraries were prepared from RNA starting material (RIN values > 7.0), using NEBNext® Ultra™ RNA Library Prep Kit for Illumina (New England Biolabs) according to the manufacturer's instructions, and library quality was checked on Agilent 2100 Bioanalyzer. Sequencing was carried out on the HiSeq 2500 (Illumina) using paired-end sequencing in Genomics, Bioinformatics and Single Cell Core, Faculty of Health Sciences, University of Macau.

### Gene set enrichment analysis (GSEA) and EMT score analysis

GSEA was performed using the R package clusterProfiler 84 with gene permutation and default parameters. GSEA was applied to selected gene sets to test their enrichment in each dataset (Figure 2D-F, Figure 7C, Figure S4D-F and Figure S7A). For polarization analysis, signature genes for featuring EMT score were selected according to previous study 85, and then EMT score was calculated using the average expression level of mesenchymal gene set minus the average expression level of epithelial gene set. Scores between positive value and negative value. Positive value means sample more like as a mesenchymal feature, negative value means sample more like as an epithelial feature.

### Clinical data analysis from The Cancer Genome Atlas (TCGA)

We chose breast cancer (BRCA). Transcriptome data (raw gene read counts) with clinical information were downloaded from GDC. Downloaded data were assembled into a matrix using R package TCGAbiolinks 86. Only genes with at least 1 count per million (cpm) or TPM value in at least 20% of total number of samples in each cohort were kept via the edgeR package or in-house scripts. Selected genes were normalized by Trimmed Mean of M-values (TMM) For further downstream analysis. For pan-cancer survival analysis, RNA-seq data across 18 cancer types were downloaded from GEO (GSE62944), this dataset provides the expression values.

### Microarray analysis

Microarray data were downloaded from the NCBI database GEO (Gene Expression Omnibus). There are *in-vitro* data from GSE77515 (Cisplatin), GSE84863 (Cisplatin/Paclitaxel), GSE17708 (TGFβ), *in-vivo* data from GSE15622 (Carboplatin/Paclitaxel). Raw intensity data were normalized by the Robust Multi-array Average (RMA) method using the 'affy' package in R-Bioconductor. Normalized data were performed in R-Bioconductor using 'limma' package to identify differentially expressed genes between different treatment samples and control samples at each time point 87, 88. Relative changes in treated versus untreated cells were expressed as base 2 logarithm of the ratio (log2FC) and only those transcripts with log2FC > 0.58 or < -0.58 and an adjusted p-value < 0.05 were considered as a significant differentially expressed genes in each group.

### Statistical analysis

Standard statistical tests including Student's t test, Wilcoxon rank sum test, Fisher exact test, Log-rank test and Cox proportional hazard regression were used for the analysis of clinical data and genomics data. Values of *P* < 0.05 were considered as statistically significant. Meta-analysis was performed using R package 'metafor'. GSEA was performed using Bioconductor package 'clusterProfiler'. Differential expression was determined using lmFit and eBayes function of 'limma' package. Correlation was studied by Pearson's correlation test as indicated. Statistical analysis was performed using R Statistical Software (version 3.4.1; R Foundation for Statistical Computing, Vienna, Austria).

### Data Availability

Microarray data were indicated in Methods. ChIP-seq data was obtained from NCBI under GEO accession no. GSE74355. The RNA-seq data in this paper are available at the NCBI under Project ID: PRJNA679982. Software and packages used in bioinformatic and statistical analysis are shown in Methods. Other data in this paper can be requested from the corresponding author.

## Supplementary Material

Supplementary figures, tables 1 and 3.

Supplementary table 2.
