# Supplementary material for: Cisplatin prevents breast cancer metastasis through blocking early EMT and retards cancer growth together with paclitaxel
Source: Theranostics. 2021 Jan 1;11(5):2442–59. doi: 10.7150/thno.46460 (PMC7797698; doi:10.7150/thno.46460)
Supplement: Supplementary file 1 — Supplementary figures, tables 1 and 3. [file thnov11p2442s1.pdf]

Cisplatin prevents breast cancer metastasis through blocking early EMT and retards cancer growth together with paclitaxel

Haitao Wang[1,2,Φ,#], Sen Guo[1,2,Φ], Seung-Jin Kim[3], Fangyuan Shao[1,2], Joshua Wing Kei Ho[4], Kuan Un Wong[1,2], Zhengqiang Miao[1,2], Dapeng Hao[1,2], Ming Zhao[1,2], Jun Xu[1,2], Jianming Zeng[1,2], Koon Ho Wong[1,5], Lijun Di[1,2], Ada Hang-Heng Wong[1,2], Xiaoling Xu[1,2], Chu-Xia Deng[1,2,*]

1. Cancer Center, Faculty of Health Sciences, University of Macau, Macau SAR, China.
2. Center for Precision Medicine Research and Training, University of Macau, Macau SAR, China.
3. Genetics of Development and Disease Branch, 10/9N105, National Institute of Diabetes, Digestive and Kidney Diseases, National Institutes of Health, Bethesda, MD, 20892, USA.
4. School of Biomedical Sciences, Li Ka Shing Faculty of Medicine, The University of Hong Kong, Hong Kong SAR, China.
5. Institute of Translational Medicine, Faculty of Health Sciences, University of Macau, Macau SAR, China.

[Φ] Authorship note: Haitao Wang and Sen Guo contributed equally to this work.

* To whom correspondence should be addressed: Chu-Xia Deng, PhD, Email: cxdeng@um.edu.mo, Tel: (853) 8822-4997, Fax: (853) 8822 2314

Running title: Cisplatin prevents breast cancer metastasis

[#] Center for Cancer Research, Clinical Research/NCI/NIH, Bethesda, MD 20892.

Supplementary Material

Supplementary Figures

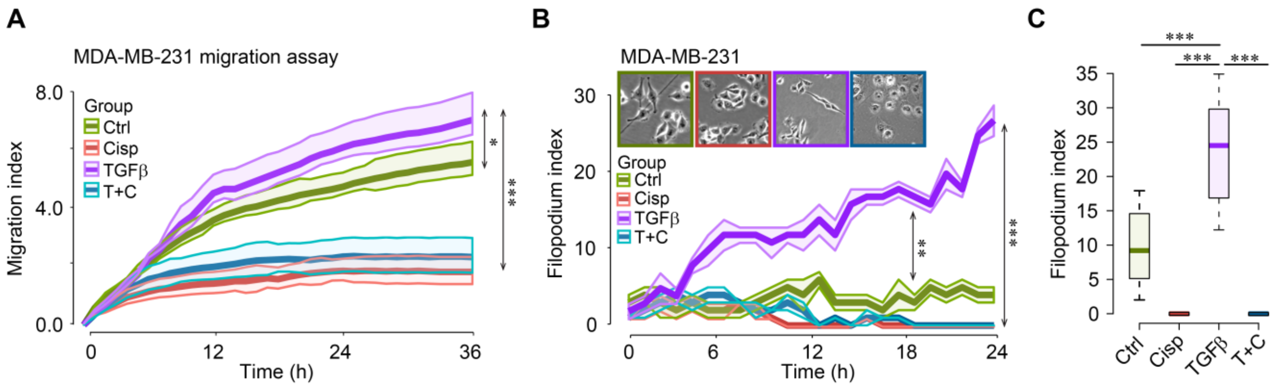

Figure S1. Cisplatin antagonizes TGFβ-induced EMT and cell movement of MBA-MD-231 cells. **A** TGFβ enhances cell migration as compared to control cells whereas cisplatin not only inhibits cell migration, but also overrides the stimulatory effect of TGFβ. **B**, **C** Calculation of filopodium index during a 24 h time lapse (**B**) and at 24h point (**C**) reveals that TGFβ significantly increases filopodium formation as compared to control, whereas cisplatin completely blocks filopodium formation in either cisplatin or cisplatin/TGFβ treatment condition in MBA-MD-231 cells. Insert in (**B**) shows cell morphology at 24 h point. In (**A-C**), data represent means ± standard deviations (SDs). Concentration of drugs in this figure: TGFβ [5 ng/mL] cisplatin [10 μM]. ns, ∗, ∗∗ and ∗∗∗ means not significant, $p < 0.05$, $p < 0.005$, and $p < 0.0005$, respectively.

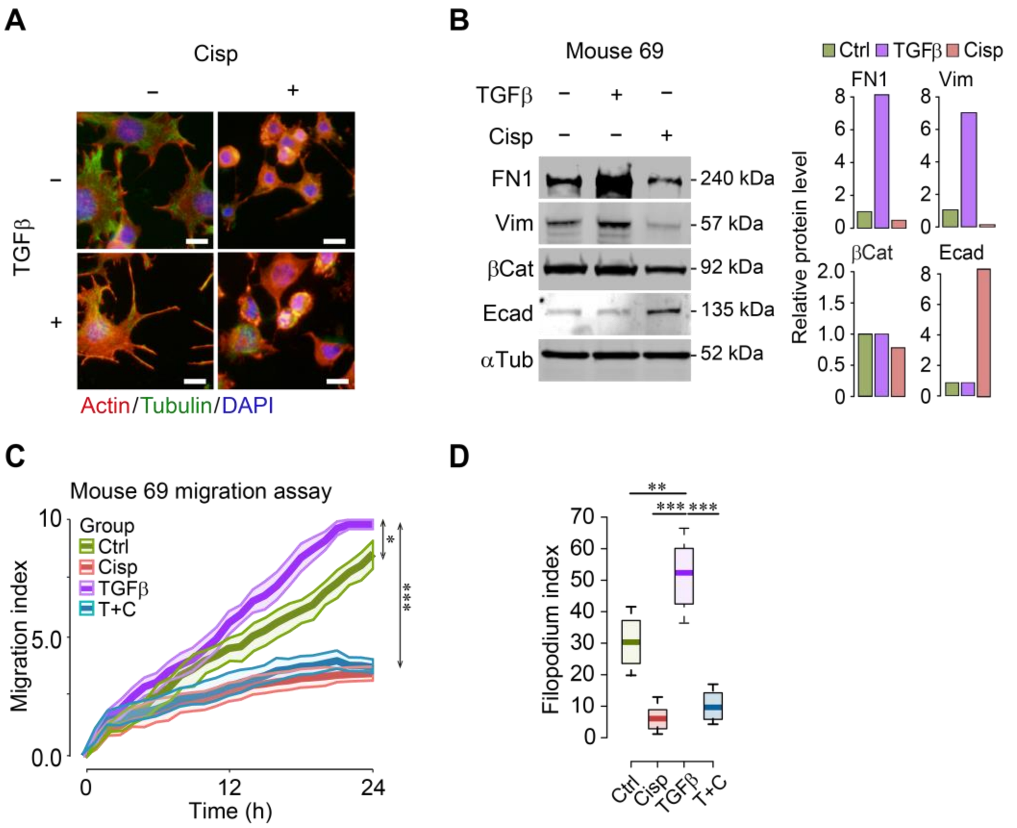

Figure S2. Cisplatin antagonizes TGFβ-induced EMT and cell movement of 69 cells. A TGFβ induces EMT in 69 cells, which is counteracted by cisplatin. Filamentous actin was stained by 1:1000 phalloidin. Bar = 50 μm. **B** TGFβ and cisplatin induce expression changes of mesenchymal and epithelial markers triggered in opposite directions revealed by Western blot. Quantification of FN1, Vimentin, β-catenin and E-cadherin levels were shown on the right panels. Replicate = 3. **C** TGFβ enhances cell migration as compared to control cells whereas cisplatin not only inhibits cell migration, but also overrides the stimulatory effect of TGFβ. **D** Filopodium index at 24 h point reveals that TGFβ significantly increases filopodium formation as compared to control, whereas cisplatin completely blocks filopodium formation in either cisplatin or cisplatin/TGFβ treatment condition in 69 cells. In (**C**, **D**), data represent means ± standard deviations (SDs). Concentration of drugs in this figure: TGFβ [5 ng/mL] cisplatin [10 μM]. ns, *, ** and *** means not significant, $p < 0.05$, $p < 0.005$, and $p < 0.0005$, respectively.

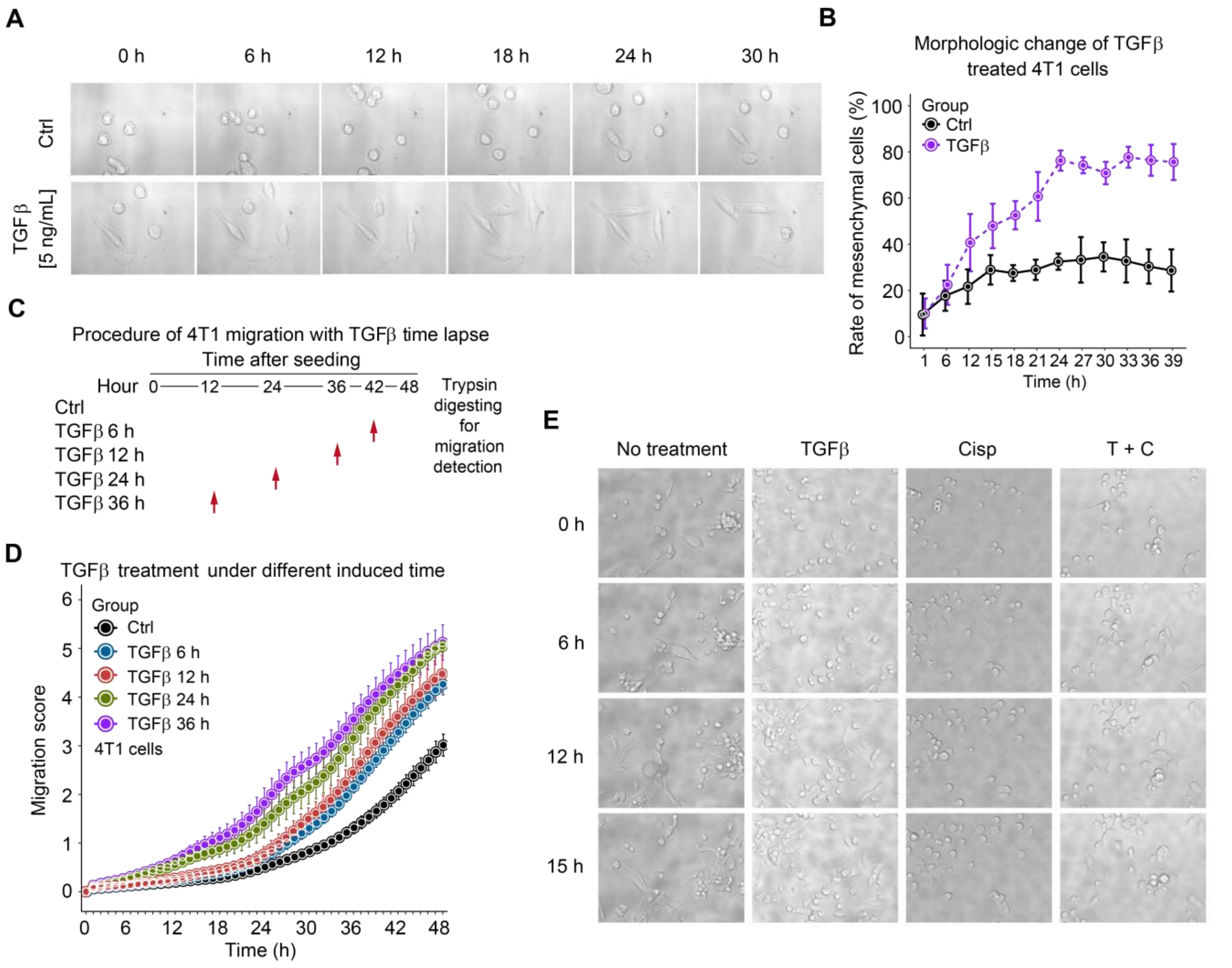

Figure S3. TGFβ induces EMT and cell movement of 4T1 cells. A, **B** 4T1 cells were treated with TGFβ and/cisplatin for a 39-h time-lapse. TGFβ induces morphologic changes of 4T1 cells into further mesenchymal status in a 39-h time lapse. Bright viewed images of cells' morphology (**A**) and summary of morphologic changes of TGFβ treated cells (**B**) were shown, which present the process of EMT. **C** A procedure of studying 4T1 cell migration stimulated by TGFβ treatment for 6, 12, 24 and 36 h. RTCA (Real-Time Cell Analysis) assay (PMID: 23094027) was conducted at 48 h after the inoculation of cells. **D** A time-course comparison of 4T1 cells treated with TGFβ, cisplatin and TGFβ/cisplatin for varying duration as indicated. At 15 h point, images showed more features of mesenchymal status compared with control group, while cisplatin treatment blocked morphological transition both with and without TGFβ treatment. **E** 4T1 cells were treated with TGFβ and/or cisplatin and subject to time-lapse imaging. TGFβ induces morphologic changes of 4T1 cells into further mesenchymal status at early times, while cisplatin [3 μM] blocks it. Brightfield images taken at four

time points of 0, 6, 12 and 15 h, respectively, were shown. In (**B**, **D**), data represent means ± standard deviations (SDs). Concentration of drugs in this figure: TGFβ [5 ng/mL]. ns, *, ** and *** means not significant, $p < 0.05$, $p < 0.005$, and $p < 0.0005$, respectively.

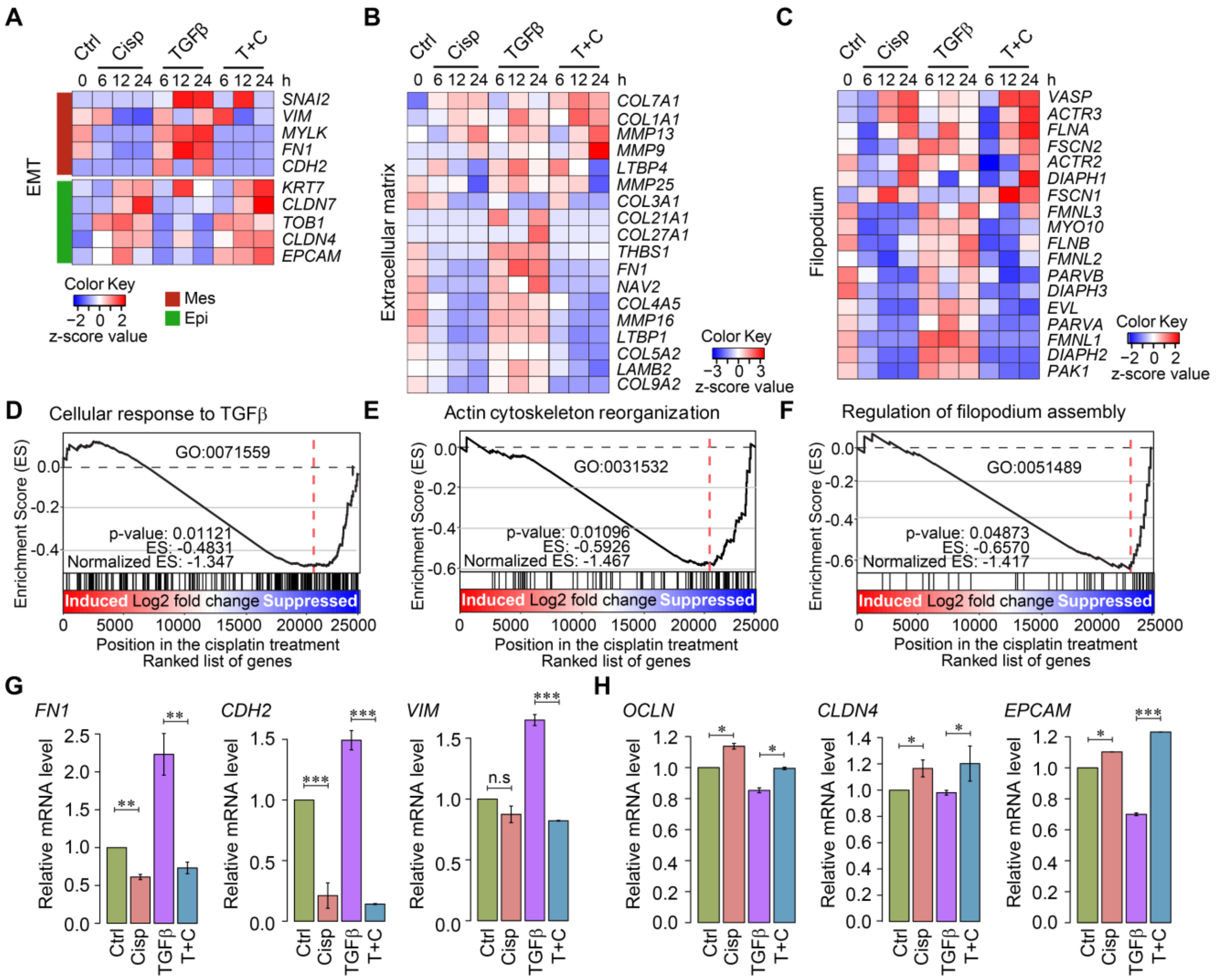

Figure S4. Bioinformatics analysis of RNA-seq data of MDA-MB-231 cells at different time points. A-C Heatmap of overall gene expression in cisplatin and/or TGFβ treated MDA-MB-231 cells showing expression changes in EMT (**A**), Extracellular matrix (**B**) and Filopodium (**C**) pathways. **D-F** Illustration of pathways related to Cellular response to TGFβ (**D**), Actin cytoskeleton reorganization (**E**) and Regulation of filopodium assembly (**F**) enriched by cisplatin treatment at 12 h point. **G**, **H** RT-qPCR validation of selected mesenchymal marker genes (**G**) and epithelial marker genes (**H**) after cisplatin and TGFβ treatment in MDA-MB-231 breast cancer cell line. In (**G**, **H**), data represent means ± standard deviations (SDs). Concentration of drugs in this figure: TGFβ [5 ng/mL] cisplatin [10 μM]. ns, ∗, ∗∗ and ∗∗∗ means not significant, $p < 0.05$, $p < 0.005$, and $p < 0.0005$, respectively.

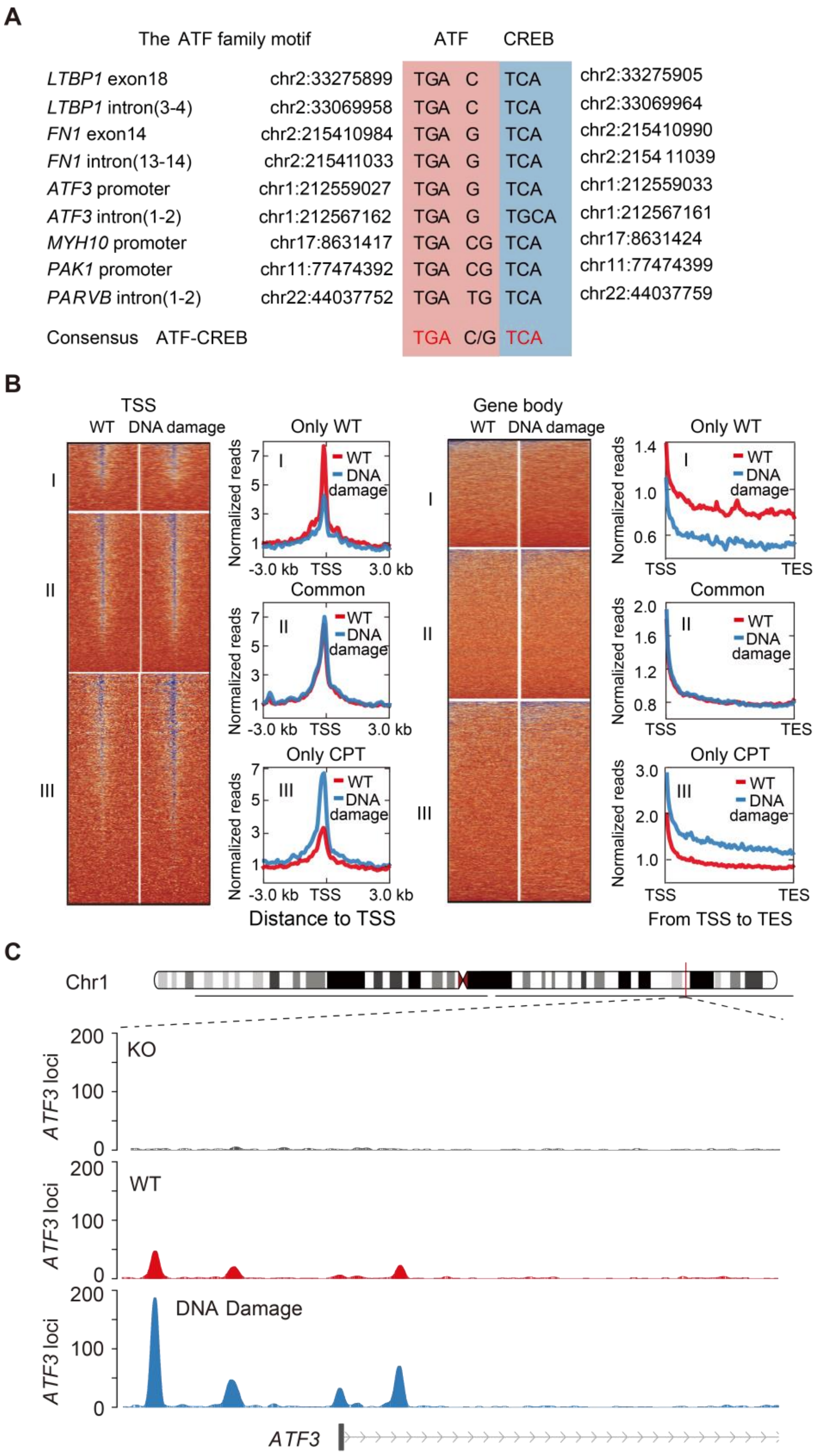

Figure S5. Transcriptional regulation of ATF3 to its downstream genes. A Consensus ATF binding site in the promoter or gene body of its downstream genes. **B** ChIP-seq reanalysis of ATF3 binding sites under normal and DNA damaging conditions in transcriptional start sites (TSS) and gene body regions. CPT treatment markedly increased binding of ATF3 to these sites. **C** ChIP-seq

analysis results showed that ATF3 binds to several loci within the *ATF3* gene and the binding is enhanced upon DNA damaging, while the binding was absent in ATF3-KO cells. ns, *, ** and *** means not significant, $p < 0.05$, $p < 0.005$, and $p < 0.0005$, respectively.

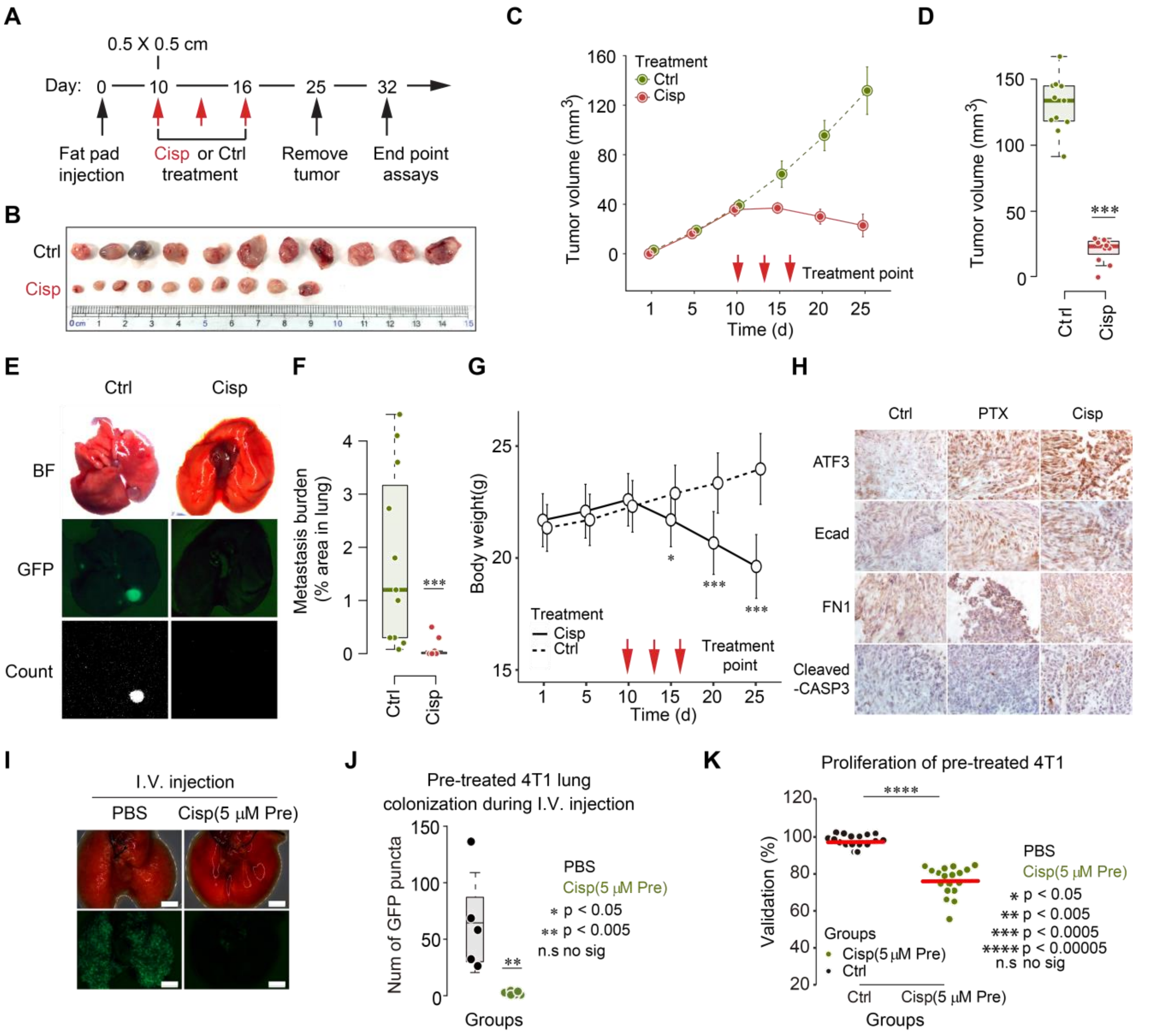

Figure S6. Cisplatin blockade of cancer metastasis in nude mice. A A procedure for studying effect of cisplatin on tumor growth and metastasis. 1×10^6 GFP-labeled 4T1 cells, which were previously trained for lung metastasis, were implanted into the mammary fad pad of BALB/c mice. When tumors reached about 0.5 cm in diameter on the average of 10 days after implantation, we treated the host mice with a dose of cisplatin at 6 mg/kg for 3 times in 9 days and tumor growth and metastasis were monitored as indicated. **B-D** Sizes and volumes of tumors during the course and the end of the treatment. **E-F** Measurement of metastasis on lungs in control and cisplatin treated mice and quantification. **G** Body weight of control and cisplatin treated mice during the 25-day experiment period. **H** IHC staining on slides of primary tumors with antibodies of cleaved-CASP3, ATF3, FN1 and E-cadherin under different treated conditions. **I** Images of colonized lungs by pre-trained 4T1

cells with/without cisplatin [5 μM] pre-treatment by dissection. Bar = 3 mm. **J** Summary and comparison of number of GFP puncta of colonized lungs by pre-trained 4T1 cells with/without cisplatin [5 μM] pre-treatment (i.v.) groups. **K** Comparison of effects of PBS and cisplatin [5 μM] pre-treatment on proliferation. In (**C**, **D**, **F**, **G**, **J** and **K**), data represent means ± standard deviations (SDs). ns, ∗, ∗∗ and ∗∗∗ means not significant, $p < 0.05$, $p < 0.005$, and $p < 0.0005$, respectively.

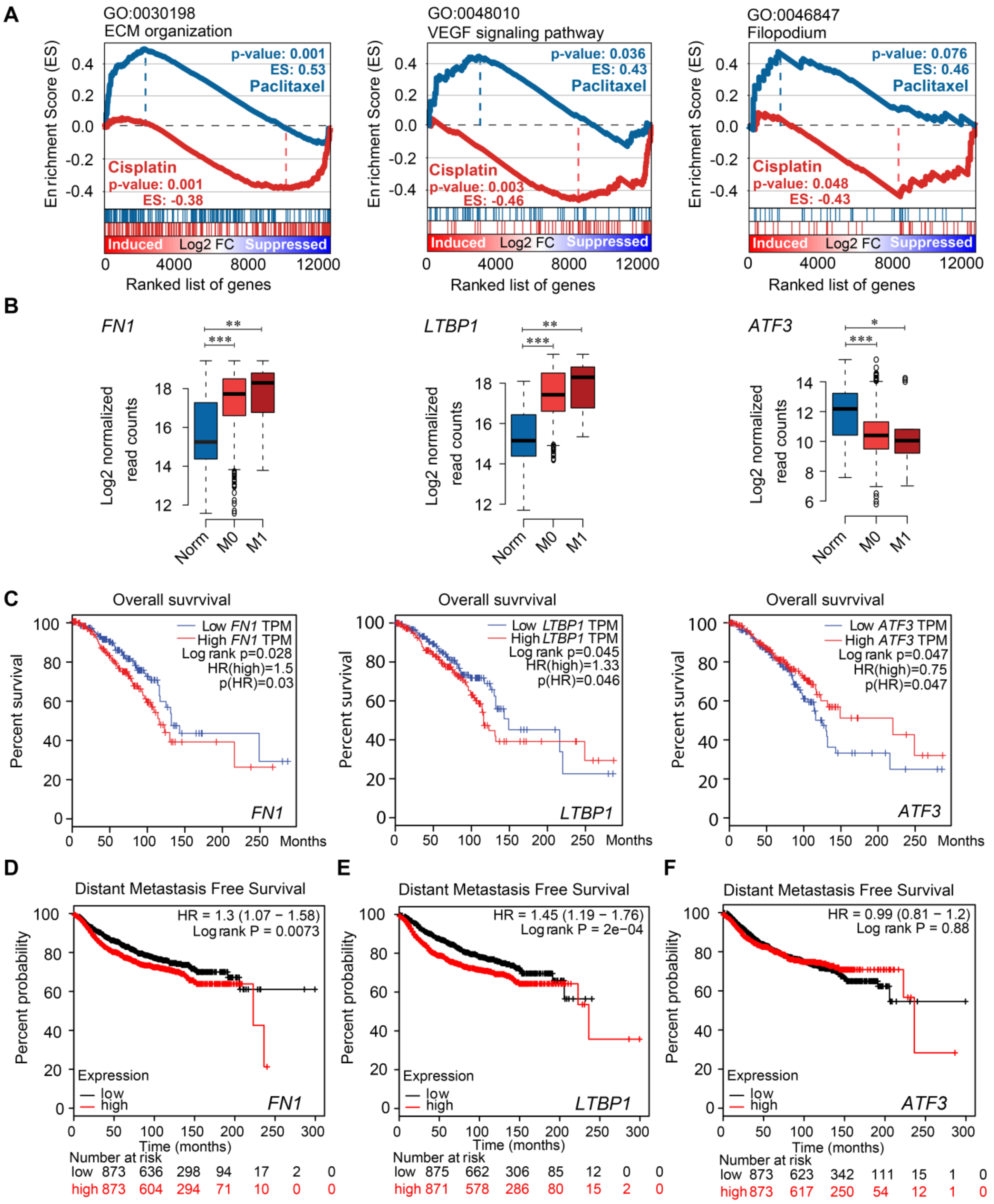

Figure S7. Bioinformatics analysis of transcriptional regulation pathways regulated by cisplatin under TGFβ treatment. A GSEA enrichment scores of ECM Organization genes (GO: 0030198), VEGF Signaling Pathway genes (GO: 0048010) and Filopodium genes (GO: 0046847) are different under cisplatin and paclitaxel treatments. FC (Fold Change). **B** Big data (TCGA BRCA 1097 samples) analysis showed that FN1 and LTBP1 are upregulated in metastatic cancers, whereas ATF3 is

downregulated in metastatic cancers. **C** Pan cancer TCGA Meta-data analysis of FN1, LTBP1 and ATF3 expression with cancer prognosis, showing that higher expression of FN1 and LTBP1 have better Overall Survival (OS) compared with lower expression, while higher expression of ATF3 have worse OS compared with lower expression, high and low expression were grouped by half of cohort. **D-F** Pan cancer KM Meta-data analysis of FN1, LTBP1 and ATF3 expression with cancer prognosis, showing that in patients with metastatic tumors, the expression levels of FN1 and LTBP1 are higher than patients without metastatic tumors, while the expression level of ATF3 is lower, high and low expression were grouped by half of cohort. In (**B**), data represent means ± standard deviations (SDs). ns, $*$, $**$ and $***$ means not significant, $p < 0.05$, $p < 0.005$, and $p < 0.0005$, respectively.

Supplementary Tables

Table S1. Antibodies used in Western blot, immunofluorescent staining and IHC.

ANTIBODIES	SOURCE	IDENTIFIER
Rabbit E-cadherin (24E10)	Cell Signaling Technology	Cat# 3195
Rabbit Vimentin (D21H3) XP mAb		Cat# 5741
Rabbit β-catenin (D10A8) XP mAb		Cat# 8480
Rabbit Rac1/2/3		Cat# 2465
Rabbit Filamin A		Cat# 4762
Rabbit ROCK1 (C8F7) mAb		Cat# 4035
Rabbit Phospho-Cofilin (Ser3) (77G2) mAb		Cat# 3313
Rabbit Phospho-Histone H2A.X (Ser139)		Cat# 2577
Rabbit α-Smooth Muscle Actin (D4K9N) XP mAb		Cat# 19245
Rabbit Lamin A/C		Cat# 2032
Anti-rabbit IgG, HRP-linked Antibody		Cat# 7074S
Anti-mouse IgG, HRP-linked Antibody		Cat# 7076S
Snail (C15D3) Rabbit mAb		Cat# 3879
Rabbit Cleaved Caspase-3 (Asp175)		Cat# 9661
Purified Mouse Anti-Fibronectin	BD	Cat# 610077
Mouse Anti-Fibronectin antibody [IST-9]	Abcam	Cat# ab6328
Rabbit Anti-ATF3 antibody [EPR19488]		Cat# ab207434
Rabbit Anti-SMAD3 antibody - ChIP Grade		Cat# ab28379
Rabbit Anti-SMAD3 (phospho S423 + S425) antibody [EP823Y]		Cat# ab52903
Anti-N Cadherin antibody		Cat# ab76057
Mouse SMAD4 (B-8)	Santa Cruz	Cat# sc-7966
Goat p-SMAD2/3 (Ser 423/425)		Cat# sc-11769
Mouse α Tubulin (TU-02)		Cat# sc-8035
Twist (Twist2C1a)		Cat# sc-81417
GAPDH (0411)		Cat# sc-47724
Monoclonal Anti-α-Tubulin antibody produced in mouse	Sigma-Aldrich	Cat# T5168
Monoclonal Anti-β-Actin antibody produced in mouse		Cat# A5316
Immobilon Western Chemiluminescent HRP Substrate	Millipore	Cat# WBKLS0500
Clarity Western ECL Substrate, 500 mL	Bio-Rad	Cat# 1705061
Animal-Free Blocker (5x) 250 mL	Vector Laboratories	Cat# SP-5030

Table S2. Sequences of qRT-PCR primers, ChIP PCR primers and shRNAs.

(In individual excel file)

Table S3. RNA expression of βActin and αTubulin during treatments.

Treatment	Time/h	ACTB	TUBA1A	TUBA1B	TUBA1C
Ctrl	0	11.5858702	6.361989356	9.393422983	8.278228697
Cisplatin	6	11.17945624	6.889456639	9.240759268	8.219981514
	12	11.32853418	6.972961035	8.424061017	8.267031925
	24	11.5858702	7.095300886	8.459578356	8.138024818
TGFβ	6	11.54025468	6.420620001	9.21757823	8.186601849
	12	11.79541855	6.526385967	8.586419558	7.928507039
	24	11.5858702	6.741829823	9.023021724	8.165091748
T + C	6	10.91716561	6.94330611	9.21757823	8.254088251
	12	11.23538619	7.189772178	8.482419955	8.398987567
	24	11.79541855	7.297573089	8.623030044	8.459578356

(TPM normalization values)
